# Game-Theoretic Obfuscation of Wi-Fi MAC-Layer Traffic Against IoT Device Fingerprinting Attacks

**DOI:** 10.3390/s26154690

**Published:** 2026-07-23

**Authors:** Abdulmajeed Alghamdi, Mnassar Alyami, Inad Alqurashi, Cliff C. Zou

**Affiliations:** 1 Department of Computer and Network Engineering, College of Computing, Umm Al-Qura University, Makkah 24382, Saudi Arabia; ayalghamdi@uqu.edu.sa; 2 Department of Electrical and Computer Engineering, University of Central Florida, Orlando, FL 32816, USA; 3 Department of Computer Sciences, College of Engineering and Computer Sciences, Jazan University, Jazan 45142, Saudi Arabia; malsaad@jazanu.edu.sa; 4 Civil Engineering Department, College of Engineering, Taif University, Taif 21944, Saudi Arabia; e.qurashi@tu.edu.sa; 5 Department of Computer Science, University of Central Florida, Orlando, FL 32816, USA

**Keywords:** IoT security, traffic fingerprinting, device identification, Wi-Fi MAC layer, cover traffic injection, traffic obfuscation, game theory, Nash equilibrium, machine learning classifiers, smart home privacy

## Abstract

Internet-of-Things (IoT) devices in smart homes are vulnerable to passive traffic fingerprinting, where an adversary captures encrypted IEEE 802.11 frames and identifies devices using MAC-layer metadata such as packet sizes and inter-arrival times. Existing defenses based on padding, traffic shaping, or synthetic cover traffic can remain vulnerable because artificial timing signatures are detectable by machine learning classifiers. This paper proposes a game-theoretic framework for evaluating Wi-Fi MAC-layer cover-traffic injection defenses. We introduce donor-based mimicry injection, in which the access point injects a replica of a paired device’s authentic traffic into each device’s stream. We compare donor mimicry with fixed-rate, exponential, and uniform synthetic baselines across 198 scenario instances (156 unique defender configurations) and eight classifiers using 10-fold cross-validation. Donor mimicry at 100% bandwidth overhead reduces the best attacker’s balanced accuracy to 33.5%, whereas synthetic methods at equal overhead reach 93.9%, showing that behavioral realism, rather than injected volume alone, drives effectiveness. Modeling the interaction as a finite two-player zero-sum game yields a mixed-strategy Nash equilibrium with game value 0.247 within the evaluated strategy space; a deployable deterministic defense holds the best pairing-unaware attacker to 25.9% balanced accuracy, near the four-class random baseline of 25%. A pairing-aware robustness analysis shows that an attacker who can orient the donor-induced identity swap recovers near-baseline accuracy, so the four-class protection presumes pairing secrecy and the durable effect is pair-level anonymity. The defense operates at the access point and requires no IoT device modifications.

## 1. Introduction

The proliferation of Internet-of-Things (IoT) devices in residential environments has created a pervasive and largely unrecognized privacy threat. Modern smart homes routinely deploy connected cameras, doorbells, lighting, and smart plugs, all communicating over Wi-Fi with the local access point (AP). While WPA2 and WPA3 encryption protect packet payloads from eavesdropping, they do not conceal MAC-layer metadata, namely frame lengths, transmission timestamps, source addresses, and inter-arrival timing, which remain visible to any passive observer within radio range [1]. A growing body of research demonstrates that these metadata features alone are sufficient for a passive adversary to identify device types, infer user activities, and profile household behavior without ever joining the network or decrypting a single frame [2,3,4].

Device fingerprinting attacks exploit the fact that each IoT device produces statistically distinctive traffic. Classifiers trained on windowed statistical features such as packet counts, size distributions, and inter-arrival statistics reliably separate these behavioral signatures even from encrypted traffic [5,6], with recent systems exceeding 95% identification accuracy [7,8] and remaining robust to basic obfuscation [9]. A passive adversary within Wi-Fi range can therefore determine which devices are active, when they are used, and by inference what the occupants are doing, all from encrypted frames. Existing defenses fall into three categories: packet padding, traffic shaping and timing perturbation, and cover-traffic injection. As Section 2 reviews in detail, all three share a fundamental limitation: they apply artificial perturbations, such as regular padding, random delays, or synthetic packet generation, whose statistical signatures are themselves detectable by modern classifiers [7,10,11]. A second limitation is the absence of formal strategic modeling: existing evaluations treat the attacker as a fixed algorithm, although a rational attacker would select the classifier that performs best against any deployed defense [12,13]. What is missing is an empirically grounded framework that jointly evaluates realistic MAC-layer traffic injection and strategic attacker–defender adaptation.

This paper develops a game-theoretic cover-traffic injection framework for Wi-Fi MAC-layer privacy. Instead of injecting artificial traffic with synthetic timing patterns, the proposed donor-based mimicry strategy injects traffic whose timing and size structure is drawn from a real paired device. By pairing devices with similar behavioral profiles and merging one device’s authentic traffic pattern with the other’s observable stream, the combined trace becomes harder to classify under pairing-unaware tabular attackers. This approach builds on the paired-device traffic shaping concept introduced by [1] and extends it through game-theoretic analysis, broader classifier evaluation, and comparison against synthetic injection baselines.

The principal contributions of this work are as follows:

- **Comprehensive obfuscation evaluation.** We evaluate four injection methods—fixed-rate, exponential, uniform, and donor mimicry—across 198 scenario instances, corresponding to 156 unique defender configurations, against eight tabular classifiers using 10-fold cross-validation.
- **Injection quality dominates quantity.** Donor mimicry reduces the best attacker’s balanced accuracy to 33.5% at ∼100% overhead, whereas synthetic methods reach 93.9% at equal overhead.
- **Game-theoretic equilibrium analysis.** We model the interaction as a finite zero-sum game and compute a mixed-strategy Nash equilibrium with game value 0.247; all equilibrium and optimality statements are relative to the evaluated classifier and configuration sets.
- **Mechanistic analysis.** We analyze donor injection through pairwise label reversal and feature-importance shifts.
- **Deployment assumptions and overhead-privacy tradeoff.** We characterize the deployment assumptions and overhead-privacy tradeoff for a trace-level AP-assisted mechanism requiring no IoT device firmware changes.

## 2. Related Work

### 2.1. IoT Device Fingerprinting Attacks

Device fingerprinting exploits the observation that each IoT device produces statistically unique traffic patterns, even when payloads are encrypted. Ref. [2] introduced behavioral fingerprinting for IoT, extracting features from network traffic to train machine learning classifiers that achieved 93–100% identification accuracy across diverse device populations. Their subsequent work [5] extended this approach to broader device categories, demonstrating that fingerprinting generalizes beyond individual device instances. Ref. [3] showed that fingerprinting remains effective on encrypted traffic, using only metadata-level features to classify IoT devices with high precision. Ref. [4] systematically evaluated multiple machine learning algorithms for traffic-based IoT fingerprinting, confirming that even simple classifiers such as Random Forest and k-Nearest Neighbors achieve reliable identification from encrypted flows.

More recent work has demonstrated that fingerprinting accuracy continues to improve despite evolving network conditions. Ref. [6] conducted a comparative analysis of six fingerprinting methods using benchmark datasets, finding that different methods excel in accuracy versus computational efficiency. Ref. [8] proposed WiFinger, a packet-level sequence matching approach for fingerprinting IoT events from noisy Wi-Fi traffic, achieving 85% recall while maintaining near-zero false positives. Ref. [14] developed DeepCRF, a deep learning approach that leverages channel state information for robust device identification even under channel variation. These advances in attack capability underscore the increasing urgency of effective defenses: as fingerprinting methods grow more precise and resource-efficient, the gap between attacker and defender widens.

At the MAC layer specifically, fingerprinting poses a particularly acute threat because 802.11 frame metadata, including timestamps, frame lengths, and source addresses, remains visible regardless of upper-layer encryption. Ref. [1] demonstrated that a passive out-of-network observer can capture encrypted Wi-Fi frames and accurately classify IoT device types using timing and size features extracted at the MAC layer. Ref. [15] extended fingerprinting to the frequency domain, showing that spectral analysis of packet timing reveals device-specific periodicities that persist across sessions. These MAC-layer attacks motivate our focus on defending at the same layer where the vulnerability originates.

### 2.2. Traffic Obfuscation and Padding Defenses

Defenses against traffic analysis broadly fall into three categories: packet padding, traffic shaping, and cover traffic injection. Each category has known limitations that our work addresses.

Packet padding modifies frame sizes to obscure size-based features. Ref. [10] evaluated eight padding methods against classifiers that use the full packet-size distribution and achieved perfect (100%) classification accuracy despite padding, demonstrating that size-only defenses are fundamentally insufficient when timing features remain exposed. Their work also proposed Dynamic STP (DSTP), which reduces per-packet overhead but still cannot prevent timing-based identification. Ref. [16] proposed random segmentation, a traffic obfuscation mechanism in which paired devices exchange dummy packets to become mutually indistinguishable, and showed that packet-size-based side-channel attacks can be mitigated, though timing-based attacks were not fully addressed.

Traffic shaping and timing perturbation methods modify inter-arrival patterns to disrupt timing-based classifiers. Ref. [17] developed stochastic traffic padding (STP) for smart homes, providing theoretical bounds on adversary detection accuracy as a function of cover traffic volume. However, STP operates at the network layer rather than the MAC layer and requires substantial bandwidth overhead. Ref. [18] proposed a dynamic dummy-traffic generation scheme that preserves contextual privacy in IoT environments, but their approach still reveals device-specific periodicity over extended observation windows. Ref. [19] introduced a differential-privacy-based traffic shaping framework, offering formal privacy guarantees but at the cost of significant latency increases that may be impractical for real-time IoT applications. Ref. [20] proposed Dynamic Traffic Padding (DTP), an adaptive approach that adjusts padding based on device types and desired privacy levels, but noted the challenge of balancing bandwidth consumption and response times. A common limitation across these methods is that adversaries can normalize injected jitter over longer observation windows, recovering stable device-specific timing profiles [7].

Cover traffic injection adds dummy packets to obscure genuine traffic patterns. Ref. [11] proposed a GAN-based dynamic obfuscation approach that generates realistic synthetic traffic, temporarily degrading classifier confidence. However, adversaries regain accuracy once models are retrained on the obfuscated traces, revealing that generated flows lack the fine-grained temporal characteristics of real device behavior. In the website fingerprinting domain, WTF-PAD [21] introduced adaptive padding that fills timing gaps with dummy packets based on statistical models of expected inter-arrival times, reducing fingerprinting accuracy with moderate overhead. More recent defenses such as FRONT [22] spread dummy packets over a wider portion of the trace, while RegulaTor [23] enforces regular transmission patterns. However, deep learning attacks have shown that these padding-based defenses can be undermined when attackers exploit residual patterns that survive the padding process [9]. These findings collectively demonstrate that synthetic or statistically modeled cover traffic is insufficient against modern classifiers that can detect the artificial nature of injected patterns.

### 2.3. Game-Theoretic Approaches to Network Security

Game theory provides a natural framework for modeling the strategic interaction between attackers and defenders in security contexts. Ref. [13] surveyed game-theoretic approaches to cyber-physical system security, demonstrating that formal equilibrium analysis can yield provably optimal defense strategies. Ref. [12] applied game-theoretic models to network defense, showing that strategic reasoning about attacker behavior leads to more effective resource allocation than heuristic approaches. Ref. [24] combined reinforcement learning with fingerprinting-based detection to select moving-target defense mechanisms against zero-day IoT attacks, demonstrating the value of adaptive, game-aware strategies. Ref. [25] evaluated adversarial attacks and defenses on both ML-based and hardware-based IoT fingerprinting systems, revealing that adversarial perturbations can significantly degrade identification accuracy while highlighting the need for robustness guarantees.

Refs. [26,27] explored adversarial machine learning for network traffic obfuscation, demonstrating that adversarial perturbations can fool flow classifiers. However, these approaches were evaluated primarily on higher-layer encrypted traffic (e.g., HTTPS) rather than Wi-Fi MAC-layer traffic generally, nor IoT-specific traces, and they do not model the interaction as a formal game with an equilibrium solution. Ref. [28] introduced TOPLDM, a dynamic packet-length distribution modification system with improved robustness under classifier retraining, but it focuses on length distributions and does not model MAC-layer timing behavior. Ref. [29] surveyed adversarial attacks on wireless sensing and fingerprinting, finding that carefully crafted perturbations can degrade accuracy, while naïve obfuscation is easily detected.

### 2.4. Positioning of This Work

Table 1 summarizes how our work relates to and extends prior contributions across three dimensions: defense mechanism, evaluation scope, and strategic modeling.

Existing traffic obfuscation defenses have three limitations relevant to this study. First, many defenses rely on artificial padding, random delays, or generated cover traffic, whose statistical signatures can remain detectable by modern classifiers [9,10]. Second, most evaluations test a small number of classifiers and do not model the attacker as a strategic agent. Third, prior game-theoretic IoT security models are usually formulated at a coarse system level rather than using measurement-driven MAC-layer timing parameters.

The work most closely related to ours is [1], which introduced paired-device traffic shaping at the MAC layer. Our framework extends this concept by evaluating donor-based mimicry against three synthetic injection baselines, using 18 statistical timing, size, and rate features, applying 10-fold GroupKFold cross-validation with temporal grouping, and formalizing the attacker–defender interaction as a finite zero-sum game with empirical minimax bounds within the evaluated strategy space.

## 3. System Model and Threat Model

### 3.1. Network Architecture

We consider a residential Wi-Fi environment in which multiple IoT devices communicate with a local access point (AP) over encrypted IEEE 802.11 traffic. The network consists of four consumer IoT devices: a smart bulb, smart plug, video doorbell, and security camera, connected to a Belkin wireless router (Belkin International, Inc., El Segundo, CA, USA). Although WPA2 protects packet payloads, it does not conceal MAC-layer metadata, including frame lengths, transmission timestamps, source and destination MAC addresses, and inter-arrival timing.

**Figure 1:**
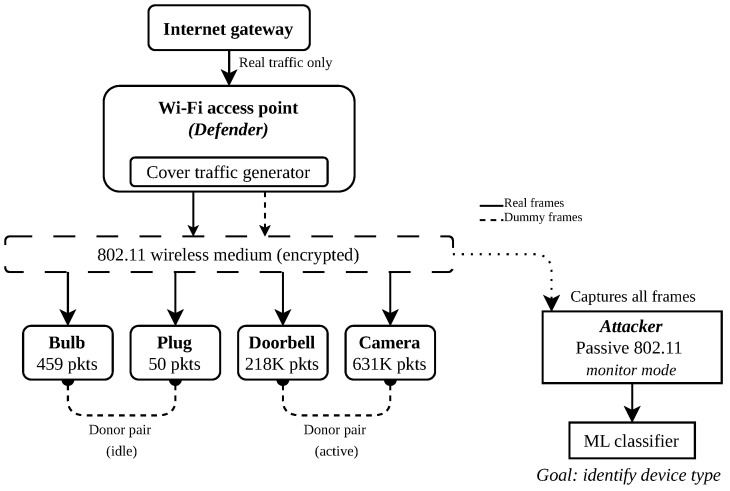
System architecture showing the network model, passive attacker, and AP-level cover traffic injection. Real frames (solid lines) and dummy frames (dashed lines) are indistinguishable to the attacker. Dummy frames are discarded by the AP before forwarding to the internet.

The defender is modeled as an AP-assisted cover-traffic mechanism with visibility into all device traffic. Figure 1 illustrates this system architecture. In the trace-level evaluation, dummy frame timestamps and sizes are merged with the protected device’s MAC-observable traffic stream before feature extraction. These dummy frames are modeled as locally generated cover frames visible to the passive monitor but not forwarded to the internet.

### 3.2. Attacker Model

We model the adversary as a passive, external observer positioned within radio range of the target AP. The attacker uses a commodity laptop with a wireless network interface card in IEEE 802.11 monitor mode to capture frames transmitted within range. The attacker does not join the target network, inject traffic, or decrypt payloads.

From the captured frames, the attacker observes MAC-layer metadata, including timestamps, frame lengths, and address fields. Address fields are used only to group frames into per-device streams and assign labels during supervised training; source and destination MAC addresses are not used as classifier input features. The attacker segments each device-associated stream into fixed-duration observation windows and computes statistical timing, size, and throughput features within each window. The evaluated feature set contains 18 statistical features: nine packet-size features, eight inter-arrival-time features, and one throughput feature.

The attacker trains a supervised machine learning classifier on labeled feature vectors, where each label corresponds to a device identity. The attacker’s objective is to correctly identify which device generated a previously unseen observation window. We evaluate eight classifiers: Random Forest, XGBoost, KNN, SVM-RBF, Logistic Regression, MLP, Gaussian NB, and Extra-Trees. The attacker is assumed to select the classifier that achieves the highest accuracy against the observed, possibly obfuscated, traffic.

### 3.3. Defender Model

The defender operates at the access point and aims to prevent the attacker from identifying individual IoT devices through traffic analysis. The defense mechanism is cover traffic injection: the AP generates additional dummy 802.11 frames and transmits them alongside the devices’ genuine traffic. These dummy frames are assumed to be indistinguishable from real frames with respect to the evaluated attacker features, namely frame timing, frame length, and per-window traffic statistics after address-based grouping.

The defender’s strategy is defined by three parameters: the injection method m∈{fixed,exponential,uniform,donor}, the observation window size w∈{1,5,10,30,60,120} seconds, and the perturbation intensity δ∈{0.0001,0.001,0.01,0.05,0.1,0.5,1.0,5.0} seconds. The injection method determines the timing distribution of dummy frames: fixed injects at a constant rate, exponential and uniform inject at randomized rates with mean δ, and donor injects using the paired device’s authentic inter-arrival times. The window size determines the temporal granularity at which the attacker observes traffic.

For donor-based injection, devices are paired according to behavioral similarity: Bulb↔Plug for idle-heavy traffic and Camera↔Doorbell for high-throughput traffic. Section 7.4 discusses donor selection when no behaviorally similar partner is available.

The defender has no knowledge of the attacker’s chosen classifier; therefore, the defense is evaluated against all classifiers in the attacker strategy set. The primary cost of the defense is bandwidth overhead, where dummy frames consume airtime on the shared wireless channel.

### 3.4. Game-Theoretic Formulation

We formalize the attacker–defender interaction as a finite two-player zero-sum game in normal form, denoted $G=\langle \left\{D,A\right\},S^{D},S^{A},u\rangle$.

**Players.** The Defender (*D*) represents the AP deploying cover traffic injection, and the Attacker (*A*) represents the passive adversary selecting a classification algorithm.

**Defender strategy space $S^{D}$.** The defender selects an obfuscation configuration defined by method *m*, observation window *w*, and perturbation intensity δ. The experimental grid contains 198 scenario instances: 3 synthetic methods × 6 windows × 8 δ values =144 synthetic scenarios, 1 donor method × 6 windows × 8 δ labels =48 donor-labeled scenarios, and 6 no-defense baselines. Because donor injection ignores δ, the 48 donor-labeled scenarios collapse to 6 unique donor configurations. Thus, the effective defender strategy space contains 156 unique configurations: 144 synthetic configurations, 6 donor configurations, and 6 baselines. The 198-row version is retained for experimental traceability, while duplicate donor rows are strategically equivalent.

**Attacker strategy space $S^{A}$.** The attacker selects one classifier from the eight evaluated algorithms, yielding $|S^{A}|=8$ pure strategies.

**Payoff function.** The payoff $u(s^{D},s^{A})$ is the mean balanced accuracy of the attacker’s classifier, computed using 10-fold GroupKFold cross-validation on the feature dataset produced by the defender’s obfuscation configuration. Balanced accuracy is used instead of plain accuracy to account for class imbalance across observation windows.

**Zero-sum property.** The attacker seeks to maximize balanced accuracy, while the defender seeks to minimize it. The defender’s payoff is therefore $1-u(s^{D},s^{A})$.

**Solution concept.** We first check for a pure-strategy equilibrium by comparing the defender’s maximin value $\min_{i}\max_{j}M\left[i,j\right]$ with the attacker’s minimax value $\max_{j}\min_{i}M\left[i,j\right]$. If they coincide, a saddle point exists. Otherwise, the mixed-strategy Nash equilibrium is computed by linear programming. The equilibrium game value $v^{*}$ represents the attacker’s accuracy under mutually optimal play of this finite empirical game. We emphasize the scope of this solution concept: all references to equilibrium, optimality, or guaranteed performance in this paper are statements about the finite strategy sets $S^{D}$ and $S^{A}$ instantiated above, estimated from one dataset and feature representation. They are not claims about unevaluated classifiers, feature sets, or injection mechanisms; Section 7.5 discusses external validity.

## 4. Methodology

**Figure 2:**
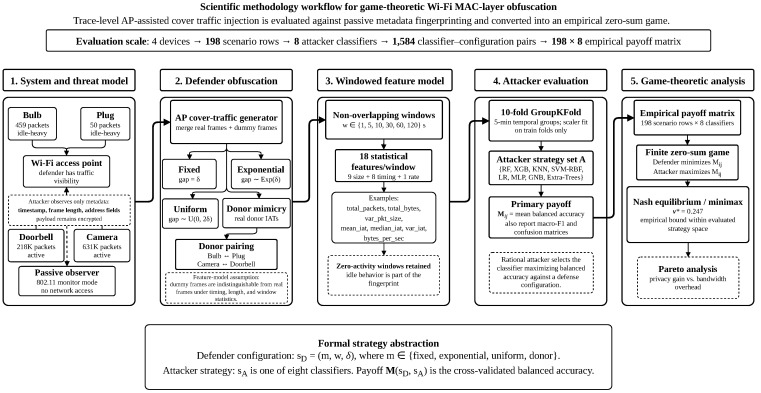
Overview of the methodology. A trace-level, AP-assisted cover-traffic injection mechanism is evaluated against a passive metadata-fingerprinting attacker and converted into an empirical zero-sum game. The workflow spans the system and threat model, defender obfuscation, the windowed feature model, attacker evaluation, and game-theoretic analysis, scaling four devices to 198 scenario rows (156 unique defender configurations, since donor injection is δ-independent) and eight attacker classifiers, yielding a 198 × 8 empirical payoff matrix of pairing-unaware balanced accuracy.

Figure 2 summarizes the experimental workflow from raw Wi-Fi captures to feature extraction, classifier evaluation, and game-theoretic analysis.

### 4.1. Data Collection and Device Characterization

We capture real 802.11 Wi-Fi traffic from four consumer IoT devices connected to a Belkin router (AP MAC addresses: 30:23:03:c2:74:9e and 30:23:03:c2:74:9f) using a monitoring station in promiscuous mode. All captures are raw 802.11 (link type 105) with WPA2 encryption. Table 2 summarizes the captured devices and their traffic characteristics. This four-device, single-environment testbed is a deliberately controlled setting: the devices span the behavioral extremes of consumer IoT (high-rate streaming versus near-idle, continuous versus sporadic), and a four-class problem constitutes a small anonymity set, the regime in which hiding a device is hardest because the attacker must separate only a handful of candidates.

The devices exhibit two distinct behavioral profiles: Bulb and Plug are idle-heavy devices with sporadic transmission, whereas Camera and Doorbell are active devices with high-throughput traffic. Donor-based obfuscation therefore pairs devices by behavioral similarity: Bulb↔Plug and Camera↔Doorbell.

### 4.2. Feature Extraction

Each device trace is segmented into non-overlapping fixed-duration observation windows of size w∈{1,5,10,30,60,120} seconds, covering short, medium, and extended attacker observation intervals.

For each window, we extract 18 statistical features from the packets contained within it. Nine features characterize the packet size distribution: total_packets (n=|P|), total_bytes ($\sum s_{i}$), unique_sizes ($\left|\{s_{i}\}\right|$), avg_pkt_size, std_pkt_size, max_pkt_size, mode_pkt_size, var_pkt_size ($\sigma^{2}$ of packet sizes), and var_size_freq (variance of the size-frequency count distribution). Eight features characterize the inter-arrival time distribution: mean_iat, median_iat, var_iat, std_iat, skew_iat, kurt_iat, min_iat, and max_iat. One rate feature, bytes_per_sec, captures the throughput. All features use standard statistical definitions (e.g., var_pkt_size = np.var(sizes), the population variance). Table 3 shows the feature definitions and categories for those statistical features extracted per observation window.

Windows containing zero packets produce all-zero feature vectors and are retained because idle behavior is part of the observable device fingerprint. Table 4 reports the zero-window prevalence by device and window size under baseline conditions.

### 4.3. Obfuscation Methods

We evaluate four cover traffic injection methods. All methods inject packets with sizes drawn from the paired donor device’s real packet size distribution, cycling through the donor’s captured packet sizes. The methods differ in the timing of injection:

**Fixed:** Packets are injected at a constant rate with inter-arrival gap =δ s. This represents Independent Link Padding (ILP), one of the oldest traffic analysis defenses.

**Exponential:** Injection gaps are drawn from an exponential distribution with mean δ, capped at 3δ to prevent outlier stalling. This models Poisson-like wireless contention patterns.

**Uniform:** Injection gaps are drawn from a uniform distribution U(0,2δ) with mean δ. This provides bounded randomization within a predictable range.

**Donor:** Injection gaps are the paired donor device’s real inter-arrival times, cycled continuously throughout the trace. Unlike the synthetic methods, donor ignores δ and injects at the donor’s natural rate, replicating its complete behavioral pattern at approximately 100% bandwidth overhead.

**Figure 3:**
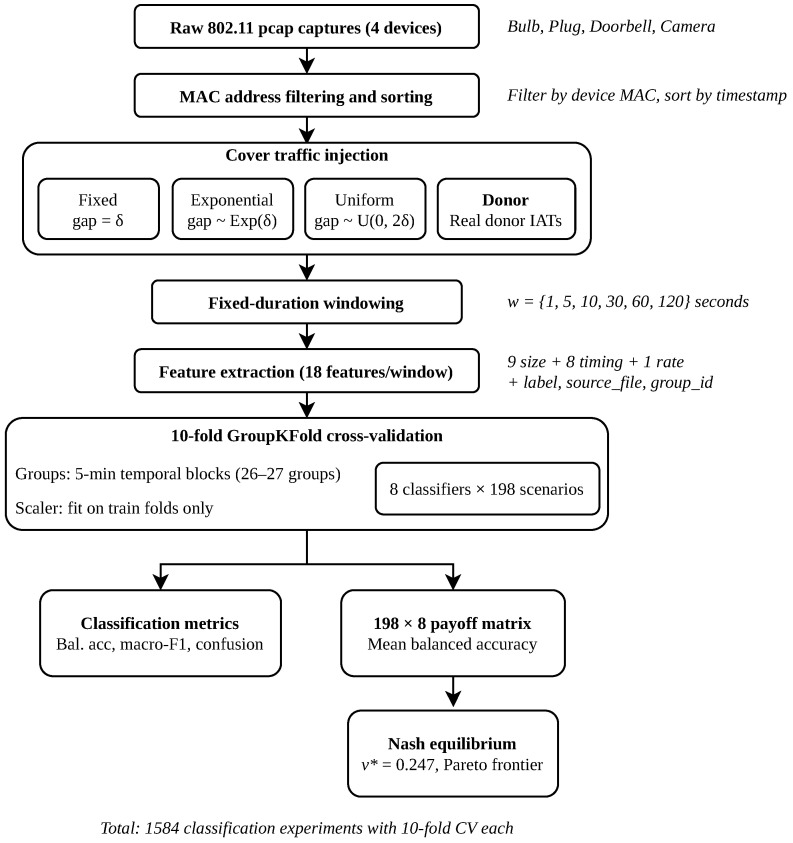
Data processing pipeline from raw 802.11 captures through cover traffic injection, feature extraction, cross-validated classification, and game-theoretic analysis. The pipeline produces 1584 classifier–configuration pairs (198 defender configurations × 8 attacker classifiers), each evaluated with 10-fold GroupKFold cross-validation, yielding 15,840 total model fits.

For the synthetic methods (fixed, exponential, and uniform), we evaluate δ∈{0.0001,0.001,0.01,0.05,0.1,0.5,1.0,5.0} s. The donor method ignores δ and uses the paired device’s natural inter-arrival sequence. Figure 3 illustrates the data processing pipeline from raw captures through game-theoretic analysis.

Injection runs continuously from the start to the end of each device trace, independent of the device’s original traffic. This process is evaluated offline on captured traces, where the merged trace approximates what the passive attacker would observe if AP-generated cover frames were visible as part of the protected device-associated stream under the evaluated feature set. The injection fills idle windows with cover traffic while leaving original packet timestamps unchanged; therefore, the defense operates through injection rather than perturbation.

### 4.4. Classification Pipeline

We model the attacker as a supervised classifier trained on windowed features. Eight classifiers spanning distinct algorithmic families are evaluated: Random Forest, XGBoost, KNN, SVM-RBF, Logistic Regression, MLP, Gaussian NB, and Extra-Trees. These classifiers represent ensemble, gradient-boosting, instance-based, kernel, linear, neural-network, probabilistic, and randomized-ensemble approaches.

All experiments were implemented in Python 3.11.4 using scikit-learn 1.8.0, XGBoost 3.2.0, NumPy 1.26.4, pandas 3.0.3, SciPy 1.17.1 (HiGHS linear programming solver), and Matplotlib 3.10.9. All hyperparameters are fixed globally across obfuscation regimes so that each classifier uses the same configuration regardless of the defense. Hyperparameters are fixed a priori based on established defaults and are not tuned per scenario. Table 5 provides the hyperparameter specifications and attacker rationale for each classifier.

Evaluation uses 10-fold GroupKFold cross-validation with temporal grouping. Each device’s trace is divided into 5-minute blocks, producing 6–7 groups per device and 26–27 groups total. GroupKFold ensures that windows from the same temporal segment never appear in both training and testing folds, preventing temporal leakage. StandardScaler is fit on training folds only and applied to test folds for classifiers that require normalization (KNN, SVM-RBF, Logistic Regression, MLP). A single random seed (42) is used for reproducibility of stochastic classifiers. All metrics are reported as mean ± standard deviation across folds. Balanced accuracy, defined as the arithmetic mean of per-class recall, is used as the primary metric to account for potential class imbalance across observation windows. Macro-averaged F1 and plain accuracy are reported as secondary metrics, while per-class precision, recall, and F1 are computed for device-specific vulnerability analysis. Although GroupKFold does not explicitly enforce class stratification, the temporal groups are approximately balanced because all four devices appear across the capture period.

## 5. Results

### 5.1. Baseline Classification on Unobfuscated Traffic

Table 6 reports the class balance across representative window sizes, confirming near-uniform distribution (22.8–26.2%) across all four devices.

Without any obfuscation, the attacker achieves high device identification accuracy across classifiers and window sizes. Table 7 shows baseline balanced accuracy for representative window sizes.

Performance varies systematically with window size. At w=1 s, classifiers achieve lower balanced accuracy because short windows contain insufficient packets for reliable statistical estimation, particularly for Bulb and Plug, which produce many zero-activity windows at this granularity. Accuracy rises through w=5 s and w=10 s, then generally plateaus at w=30–60 s. At w=120 s, accuracy slightly decreases for some classifiers due to reduced sample size.

**Figure 4:**
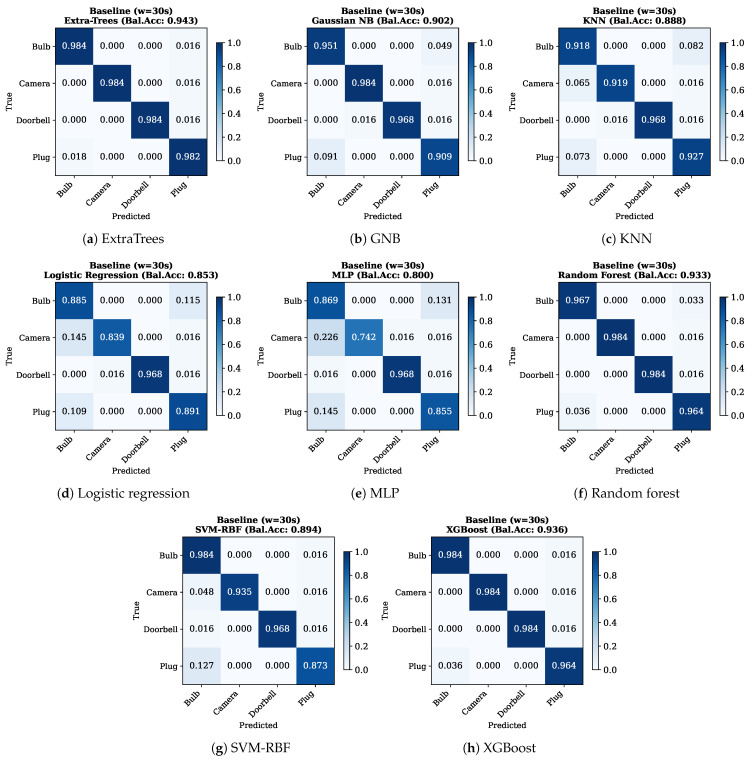
Baseline confusion matrices for all eight classifiers at w=30 s. Values represent normalized per-class recall. Misclassification is predominantly pairwise within donor pairs, Bulb↔Plug, while Camera and Doorbell are classified with near-perfect accuracy.

Confusion analysis reveals that misclassification is predominantly pairwise within donor-paired devices. At w=30 s, Random Forest confuses Bulb with Plug (Bulb→Plug: 3.3%, Plug→Bulb: 3.6%) but never confuses devices across pairs (Camera→Bulb: 0%, Doorbell→Plug: 0%). Camera and Doorbell are classified with 98.4% per-class accuracy. Figure 4 shows the confusion matrices for all eight classifiers at w=30 s.

**Figure 5:**
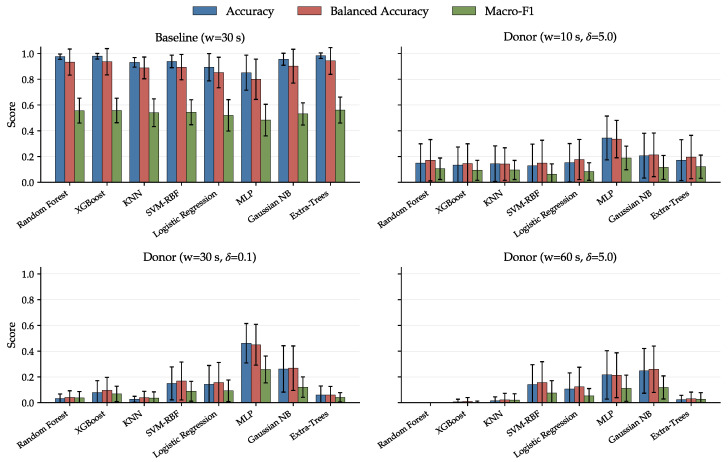
Comparison of accuracy, balanced accuracy, and macro-F1 across four scenarios: baseline (w=30 s), donor (w=10 s, δ=5.0), donor (w=30 s, δ=0.1), and donor (w=60 s, δ=5.0). All three metrics are consistent under baseline conditions and collapse together under donor injection, confirming that the defense effectiveness is not an artifact of metric choice.

To validate the choice of balanced accuracy as the primary metric, Figure 5 compares plain accuracy, balanced accuracy, and macro-F1 across all eight classifiers at baseline w=30 s. Plain accuracy and balanced accuracy are closely aligned (within 4 percentage points for all classifiers), confirming that class imbalance is moderate and does not distort accuracy-based conclusions. Macro-F1 is consistently lower than both (0.48–0.56 versus 0.80–0.94), reflecting the harmonic-mean penalty: at w=30 s, each cross-validation fold contains approximately six test samples per class, so a single misclassification substantially reduces per-fold precision or recall for that class. Balanced accuracy, which averages per-class recall without the harmonic-mean penalty, is more robust at these sample sizes and is therefore used as the primary metric throughout this paper. Under donor injection (Figure 5, remaining panels), all three metrics collapse together to near-zero, confirming that the defense effectiveness is not an artifact of metric choice.

### 5.2. Synthetic Obfuscation Methods Are Ineffective

**Figure 6:**
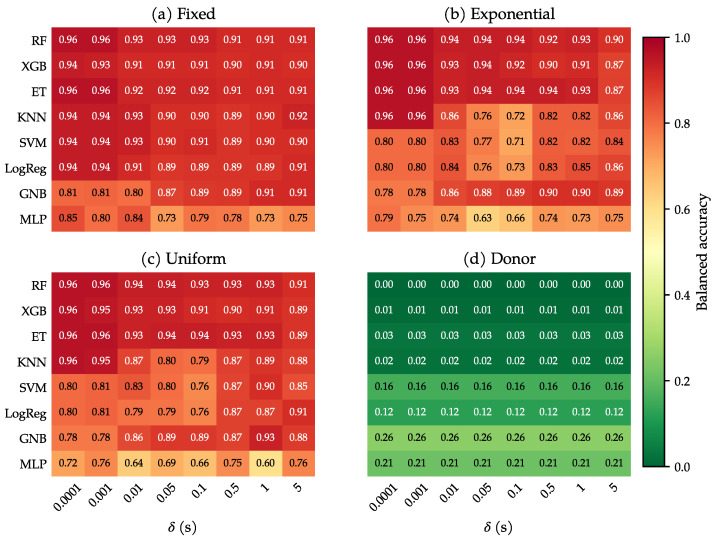
Attacker balanced accuracy by obfuscation method at the equilibrium-relevant window w=60 s: (**a**) Fixed, (**b**) Exponential, (**c**) Uniform, (**d**) Donor. Rows are attacker classifiers; columns are injection intensities δ. Synthetic methods (**a**–**c**) leave the ensemble attackers (RF, XGB, ET) at or above 0.87 at every δ and no classifier below 0.60, while donor (**d**) drives the ensembles to 0.00 and holds every classifier at or below 0.26. The identical columns in (**d**) reflect the donor method’s δ-independence; the same qualitative pattern holds at all six window sizes.

Within our evaluated MAC-layer feature/classifier setting, the three synthetic injection methods, namely fixed, exponential, and uniform, provide little to no protection against the best-response attacker. Across all 384 synthetic classifier–configuration combinations per method, the mean balanced accuracy remains 0.895±0.080 (fixed), 0.866±0.103 (exponential), and 0.872±0.100 (uniform). When considering the best attacker classifier per configuration (the rational attacker’s choice), fixed achieves a median of 0.941 (IQR: 0.913–0.965), exponential 0.940 (IQR: 0.901–0.963), and uniform 0.940 (IQR: 0.913–0.963). Of the 48 configurations per synthetic method, the best attacker exceeds 85% balanced accuracy in 95.8% (fixed), 85.4% (exponential), and 91.7% (uniform) of cases. By contrast, the donor method achieves a mean of 0.170±0.102, with the best attacker never exceeding 0.85 in any of its 48 configurations (0/48, 0.0%). Figure 6a–c visualizes this separation at the equilibrium-relevant window w=60 s; the pattern is consistent across all six window sizes.

Because all four methods draw injected packet sizes from the donor’s real size distribution (Section 4.3), any performance difference is attributable to injection timing alone; the synthetic baselines are therefore stronger than the constant-size dummy packets of prior work, yet they still fail to degrade attacker accuracy.

At small δ values (0.0001–0.001), which produce massive injection volumes (7–70 million packets, 830–8300% bandwidth overhead), attacker accuracy remains above 95%. The classifiers learn to distinguish the regular/random injection patterns from genuine device traffic and effectively filter them out. At large δ values (1.0–5.0), injection is too sparse to affect the feature distributions. The three synthetic methods produce nearly identical results at every configuration, confirming that the specific distribution of injection timing (constant, exponential, or uniform) is irrelevant. The artificial nature of the timing, not its distributional form, is what makes it detectable.

### 5.3. Donor-Based Mimicry Is Highly Effective

The donor method dramatically outperforms all synthetic methods, reducing attacker accuracy to near-random levels across all classifiers. Figure 6d shows balanced accuracy values between 0.00 and 0.26 across all classifiers and δ values at w=60 s, compared to 0.60–0.96 for the synthetic methods in panels Figure 6a–c.

The donor method achieves this by injecting a complete behavioral replica of the paired device’s traffic. Because the donor ignores δ and injects at the donor’s natural rate, all 48 donor scenarios produce similar results. At the strongest configuration (w=60 s), Random Forest achieves 0.000±0.000 balanced accuracy across all 10 CV folds, meaning no test sample is correctly classified on any fold. This occurs because the donor injection makes each device’s feature distribution nearly identical to its paired partner’s, and RF’s deterministic splitting rules consistently assign every sample to the paired device’s class rather than the true class. The confusion matrices reveal the mechanism: a perfect pairwise identity swap. Bulb is classified as Plug 100% of the time, Camera as Doorbell 97%, Doorbell as Camera 94%, and Plug as Bulb 97%. The classifier confidently assigns every device to its paired partner.

**Figure 7:**
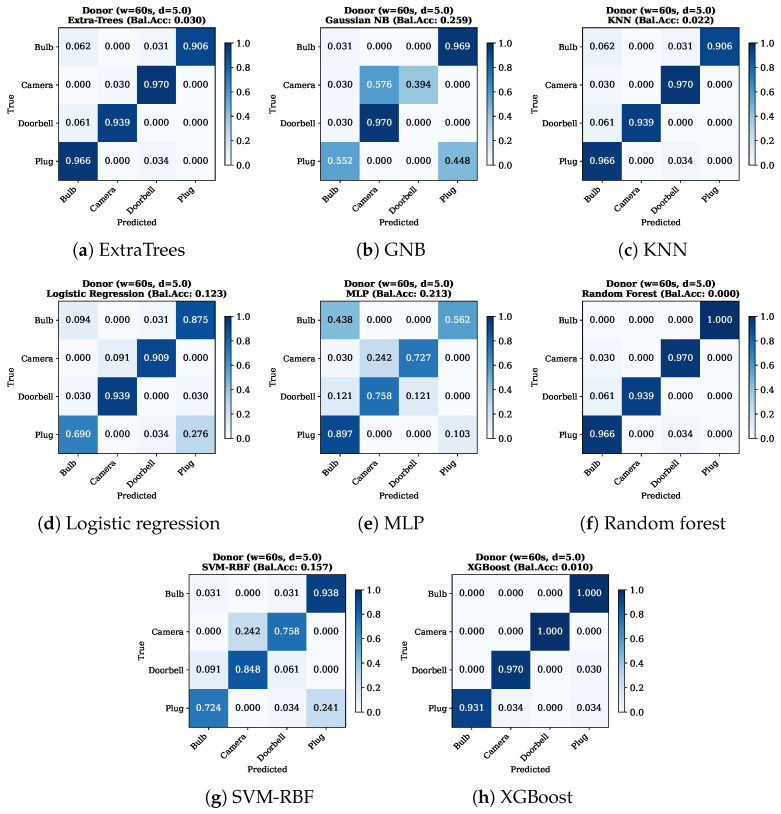
Donor confusion matrices for all eight classifiers at w=60 s and δ=5.0. Values represent normalized per-class recall.

The “balanced accuracy =0.000” result for RF at w=60 s reflects a systematic misclassification structure rather than uniformly random predictions: the classifier confidently assigns every device to its paired partner. An adaptive attacker who knows that donor pairing is deployed could in principle invert predictions within the paired sub-problem, recovering high accuracy on the “Bulb vs. Plug” and “Camera vs. Doorbell” two-class problems. The privacy benefit of donor mimicry is therefore best understood as within-pair anonymity: under donor injection, the attacker cannot distinguish Bulb from Plug or Camera from Doorbell, but can still distinguish the idle pair (Bulb, Plug) from the active pair (Camera, Doorbell) on the basis of aggregate traffic volume and rate. The 4-class balanced accuracy figures reported throughout this work are conservative in this sense: they treat each device as a distinct class and report the attacker’s success on that 4-class problem, which corresponds to a non-adaptive attacker who does not know about the pairing. Section 6.7 evaluates the pairing-aware attacker quantitatively through oracle relabeling. Figure 7 displays the corresponding confusion matrices under donor injection (w=60 s, δ=5.0), where the off-diagonal dominance confirms the pairwise identity swap.

### 5.4. Controlled Volume Comparison

To isolate the contribution of injection quality from injection quantity, Table 8 compares all methods at matched injection volume. At approximately 700,000–850,000 injected packets (∼100% overhead), the donor method achieves 33.5% balanced accuracy while fixed, exponential, and uniform achieve 93.9%, a 60-percentage-point gap at equivalent bandwidth cost.

Remarkably, increasing synthetic injection volume to 83× that of donor (70.8 million packets, 8300% overhead) paradoxically increases attacker accuracy to 95.2%, as the regular injection patterns provide additional discriminative features. This demonstrates that obfuscation effectiveness depends on the behavioral realism of injected traffic, not its volume.

### 5.5. Interaction Analysis

**Figure 8:**
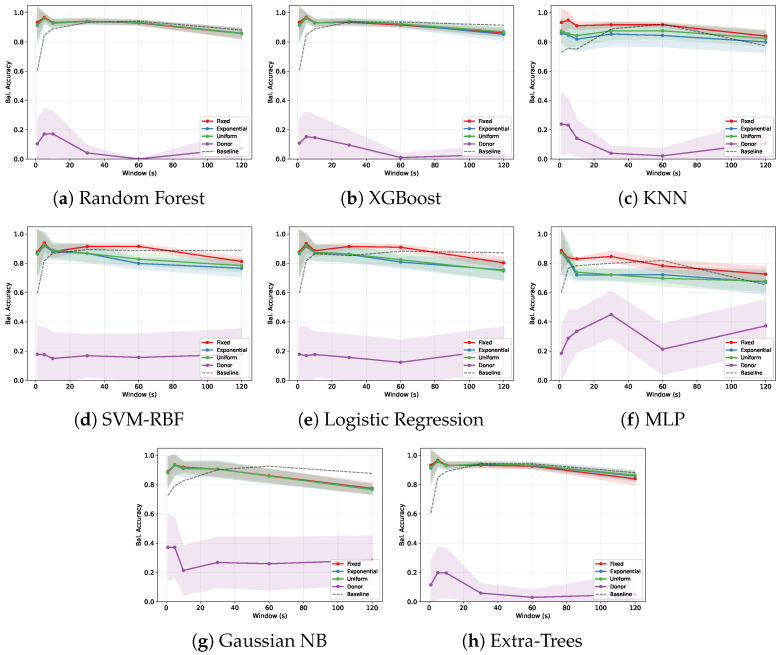
Interaction between window size and obfuscation method for each classifier. Lines represent obfuscation methods, and shaded regions indicate ±1 standard deviation across cross-validation folds. Synthetic methods generally show increasing balanced accuracy with window size, whereas the donor method generally shows decreasing balanced accuracy.

The effectiveness of obfuscation depends on the interaction between window size, injection method, and classifier choice. Figure 8 presents interaction plots showing balanced accuracy as a function of window size, with separate lines for each obfuscation method, across representative classifiers.

Two patterns emerge. First, for synthetic methods, balanced accuracy increases monotonically with window size across all classifiers. It is noticeable that longer observation windows give the attacker more packets to estimate stable feature statistics, improving classification. This trend holds regardless of the injection method or δ value, confirming that synthetic injection provides no defense even when the attacker is limited to short observation windows. Second, for the donor method, the relationship is inverted: balanced accuracy generally decreases with larger window sizes, as longer windows allow more donor packets to accumulate and dominate the feature statistics. The strongest defense occurs at w=60 s, where the donor traffic constitutes a sufficient proportion of the combined stream to induce the identity swap observed in the confusion matrices.

The interaction plots also reveal classifier-specific behavior under donor injection. Ensemble classifiers (Random Forest, XGBoost, Extra-Trees) show the steepest accuracy decline with increasing window size, dropping from 0.35–0.45 at w=1 s to near-zero at w=60 s. By contrast, MLP and Gaussian NB maintain relatively flat accuracy profiles across window sizes (0.25–0.45), reflecting their ability to exploit per-feature or nonlinear timing signals that persist regardless of observation duration. This classifier-specific interaction pattern is consistent with the Nash equilibrium, which selects GNB and MLP as the only viable attacker strategies.

### 5.6. ROC Analysis

**Figure 9:**
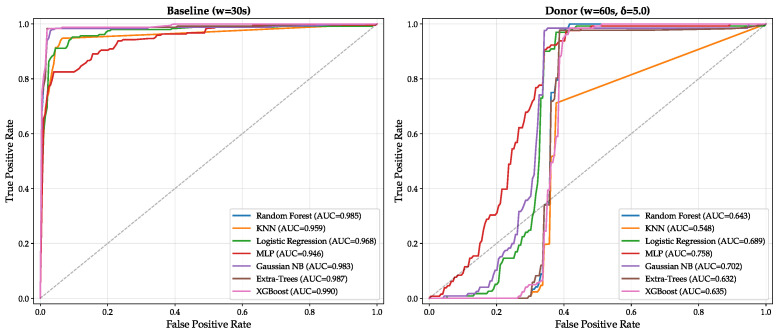
ROC curves comparing baseline (w=30 s) and donor (w=60 s, δ=5.0) conditions. AUC drops from 0.946–0.990 under baseline to 0.548–0.758 under donor injection, with KNN falling to near-random (0.548) and MLP retaining the highest residual AUC (0.758). The dashed diagonal line indicates the performance of a random classifier.

Figure 9 presents receiver operating characteristic (ROC) curves comparing baseline (w=30 s) and donor (w=60 s, δ=5.0) scenarios. Under baseline conditions, all classifiers achieve AUC values between 0.946 (MLP) and 0.990 (XGBoost), with curves hugging the upper-left corner. Under donor injection, AUC collapses to 0.548–0.758, with KNN falling to near-random (AUC = 0.548). MLP achieves the highest residual AUC (0.758), consistent with its role in the Nash equilibrium attacker strategy.

### 5.7. Feature-Level Mechanisms of Donor Injection

To understand how donor injection alters each classifier’s discriminative signal, we examine the shift in feature importance between baseline and donor regimes for the three classifiers most relevant to the equilibrium analysis: Random Forest (the strongest baseline attacker, but collapsed under donor), Gaussian NB (primary equilibrium attacker, 72.8% probability), and MLP (secondary equilibrium attacker, 27.2% probability). For Random Forest, we report impurity-based (Gini) importance averaged across the 200 trees; this measure quantifies each feature’s contribution to in-training split decisions and is well-suited to characterizing how an ensemble that exploits joint feature distributions is disrupted by the donor method. For Gaussian NB and MLP, we report permutation importance computed on test folds using balanced accuracy as the scoring metric, with 10 repeats per fold averaged across 10 GroupKFold folds. Permutation importance is the appropriate measure for non-ensemble classifiers because it captures each feature’s post-training contribution to predictive performance rather than its in-training split frequency. All importances are normalized so that each classifier’s vector sums to 1.0 across the 18 features. To separate the effects of the obfuscation method from the effects of window size, we report all twelve combinations of {classifier, window, method} for windows w∈{30s,60s}, with all donor cells labeled δ=5.0 (the donor method is δ-independent, see Section 6.1).

Table 9 and Table 10 report the per-feature importance at w=30 s and w=60 s, respectively, with baseline (BL) and donor (DN) columns for each of the three classifiers. Reporting both windows separately isolates the effect of the obfuscation method from the effect of observation duration: within each table, the BL and DN columns differ only in the presence of donor injection, and the two tables differ only in window size. Table 11 aggregates the resulting within-window shifts at the category level.

The feature-importance analysis (Table 9 and Table 10) reveals that the donor method’s mechanism is classifier-specific and window-dependent. Three patterns emerge. First, ensemble and probabilistic classifiers (RF, GNB) shift in the same direction under donor injection at both window sizes: size-feature importance decreases and timing-feature importance increases. For RF, the size loss is 9.4 pp at w=30 s and 5.4 pp at w=60 s, with corresponding timing gains of 10.1 pp and 3.4 pp. For GNB, the same direction holds but the magnitudes are larger: size losses of 18.7 pp (w=30 s) and 7.3 pp (w=60 s), and timing gains of 22.0 pp (w=30 s) and 14.4 pp (w=60 s). These shifts reflect the intended donor mechanism: injecting packets drawn from the paired donor’s real size distribution directly disrupts the size-feature category that RF and GNB rely on most under baseline. GNB additionally shows a near-total collapse of rate-feature importance under donor (w=60 s: 0.081→0.010), consistent with the fact that donor injection roughly doubles per-device byte rate and GNB’s permutation importance, which reflects post-training discriminative value, registers that the rate feature has lost its class-discriminative power.

Second, MLP exhibits a qualitatively different and window-dependent pattern. At baseline w=30 s, MLP is timing-dominant (Size =0.297, Timing =0.703), with max_iat alone contributing 0.373 of total importance. Under donor at w=30 s, MLP’s category dependence inverts: size importance rises by 15.5 pp to 0.452, while timing importance falls by 20.0 pp to 0.503. At w=60 s the picture is again different: MLP’s baseline category dependence is already much more balanced (Size =0.512, Timing =0.474), and donor injection produces only a small shift (−3.1 pp size, −1.9 pp timing). The window size therefore controls not only MLP’s vulnerability to donor injection but also MLP’s baseline feature reliance: MLP at short windows is timing-anchored and donor disrupts that anchor, whereas MLP at long windows has already partially abandoned the timing anchor and donor produces little additional shift. This window-dependence of MLP’s feature reliance is itself larger than the window-dependence of either RF or GNB (MLP’s baseline Size aggregate moves 0.297→0.512 from w=30 s to w=60 s, while RF and GNB shift by less than 0.030 across the same window change).

Third, despite these different mechanisms, all three classifiers converge under donor at w=60 s to comparably balanced feature distributions (RF: 0.514/0.402; GNB: 0.492/0.498; MLP: 0.481/0.456), with no single feature exceeding 0.12 of total importance in any donor-regime column at w=60 s. This convergence is itself the mechanistic signature of donor’s effectiveness: the defense does not target a specific feature or category, it spreads the discriminative signal thinly across the feature space such that no learning algorithm, whether one that exploits joint distributions (RF), per-feature marginals (GNB), or nonlinear combinations (MLP), recovers more than ∼30% balanced accuracy at w=60 s. The same convergence does not hold at w=30 s, where GNB retains a timing-heavy distribution (Timing =0.553) and MLP retains a balanced distribution (Timing =0.503, Size =0.452); this helps explain why MLP achieves its highest donor-regime accuracy (45.0%) at w=30 s rather than w=60 s. At the shorter window, the feature landscape is less uniformly flattened, and MLP’s nonlinear hidden layers exploit the residual structure that ensembles miss.

These shifts explain the role reversal documented later in the method × classifier analysis: RF’s baseline strength rests on interactions among the dominant size features, which donor flattens; GNB extracts residual marginal signal from individual timing features (std_iat and max_iat grow from 0.090 to 0.165 of its importance at w=60 s); and MLP composes nonlinear functions of the redistributed signal despite losing its dominant max_iat anchor (0.373 to 0.094 at w=30 s). Their partial survival aligns with the Nash equilibrium of Section 6, which selects GNB and MLP as the only supported attacker strategies.

### 5.8. Per-Device Vulnerability

**Figure 10:**
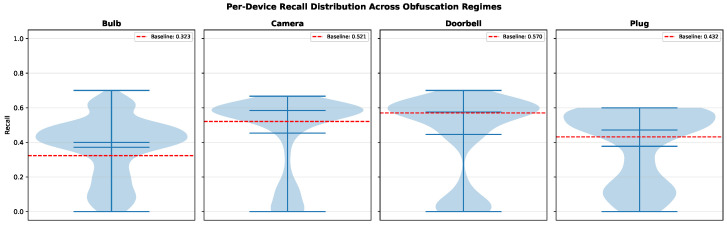
Per-device recall distributions across all obfuscation regimes. Bimodal distributions reflect the separation between synthetic configurations, high recall, right cluster, and donor configurations, low recall, left cluster. Baseline recall is indicated by reference lines.

Device vulnerability varies with behavioral profile. Doorbell is the most consistently identifiable device (median recall ∼0.60 across all obfuscation regimes), reflecting its distinctive high-rate continuous traffic pattern. Bulb is the most successfully obfuscated (median recall ∼0.35), as its idle-heavy profile becomes indistinguishable from Plug under donor injection. The per-device recall distributions (Figure 10) show clear bimodality: a cluster near 0 (donor configurations where the device is unidentifiable) and a cluster near 0.5–0.7 (synthetic configurations where the device remains identifiable).

### 5.9. Factor Decomposition and Method × Classifier Interaction

Having established the overall effectiveness of donor-based mimicry, we now decompose the contribution of each experimental factor to attacker accuracy. The 1584 classifier–configuration cells of the payoff matrix span four design dimensions: obfuscation method (5 levels including baseline), observation window (6 levels), classifier family (8 levels), and injection rate δ (8 levels, applicable to synthetic methods only). Table 12 reports the marginal mean balanced accuracy and macro-F1 along each dimension, averaging across all other dimensions.

Two interpretive cautions apply. The dispersion column reports cell-to-cell variation across each marginal, not per-cell cross-validation variance; it is large for the Window-size and Classifier marginals because each mixes donor cells (≈0.17) with synthetic cells (≈0.88). The δ marginal is restricted to synthetic methods, since donor injection is δ-independent, and each marginal cell is an unweighted mean of its constituent cells.

The marginal analysis identifies the obfuscation method as the dominant factor by a wide margin. Donor-based mimicry reduces mean balanced accuracy by 72.5 percentage points relative to the best synthetic method (fixed, 0.895→0.170). By contrast, window size shifts accuracy by only 10.2 points across its range (0.648 at w=120 s to 0.750 at w=5 s), classifier choice by 7.5 points (0.661 for MLP to 0.736 for Gaussian NB), and δ by 12.2 points within synthetic methods (0.807 at δ=5.0 to 0.929 at δ=0.001). The Method marginal is also the only one where the within-factor dispersion is small relative to the between-level spread, indicating that the method choice produces a robust separation. The large dispersion values in the Window-size and Classifier marginals (∼0.30–0.36) reflect the bimodal distribution of cells: each window size and each classifier appears in both donor and synthetic regimes, and the gap between these regimes dominates within-marginal variability. This is itself an informative observation. That is, no single classifier or window size, in isolation, distinguishes the two regimes; only the method does.

The marginal decomposition cannot reveal the interaction between method and classifier, which is the most consequential finding of this section: the ranking of classifiers reverses under donor injection. Table 13 reports the full method × classifier mean balanced accuracy, exposing this reversal directly.

Two findings dominate Table 13. First, the strongest attacker under baseline and all synthetic methods is an ensemble classifier (XGBoost or Random Forest, 0.855–0.927 mean balanced accuracy), with MLP consistently the weakest. Second, under donor injection, this ranking inverts: MLP becomes the strongest attacker (0.307), Gaussian NB second (0.294), and the ensemble classifiers (RF and XGBoost) become the weakest (0.090–0.093). Extra-Trees, despite being a near-twin of Random Forest, collapses to 0.108. The two best baseline attackers are the two worst donor attackers, and vice versa.

This reversal is mechanistic, not statistical. Ensemble classifiers achieve their baseline strength by exploiting interactions between size-based and timing-based features (e.g., the joint distribution of var_pkt_size and var_iat distinguishes Bulb from Plug even when each marginal looks similar). Donor injection merges these joint distributions across paired devices: under donor, the joint distribution of (sizes, timings) for Bulb-with-injection is statistically equivalent to that of Plug-with-injection, because the injection itself supplies Plug-like packets at Plug-like times. Tree-based methods that learned to discriminate on the joint distribution become confidently wrong, producing the near-zero balanced accuracy and the pairwise identity swap observed in Figure 7. MLP and Gaussian NB partially resist for distinct reasons. Gaussian NB models each feature independently, so even partial survival of marginal discriminability in some features (e.g., min_iat, which the donor injection cannot drive to zero) provides residual classification signal. MLP’s nonlinear hidden layers can in principle capture residual timing structure that ensemble methods do not encode as splits, though even MLP achieves only 30.7% mean balanced accuracy under donor, only modestly above the 25% floor. This is consistent with the Nash equilibrium of Section 6, which selects Gaussian NB and MLP as the only attacker strategies receiving positive probability.

The role-reversal also has a practical implication for prior fingerprinting studies. Evaluations that test new defenses against a single state-of-the-art classifier, typically Random Forest or XGBoost, risk drawing the wrong conclusion when the defense is of the donor-mimicry type. For such defenses, the strong classical attackers collapse to near-zero accuracy, but weaker-on-average classifiers like MLP retain residual capability. A defense evaluation that does not include both ensemble and non-ensemble classifiers in its attacker model can either overstate or understate effectiveness depending on which class of attacker happens to be tested. Our eight-classifier evaluation makes this trade-off visible and motivates the game-theoretic formulation in Section 6, which treats classifier selection as a strategic decision rather than a fixed experimental setting.

## 6. Game-Theoretic Analysis

This section instantiates the game model defined in Section 3.4 with the empirical payoff matrix derived from the 1584 classifier–configuration pairs reported in Section 5. We compute the Nash equilibrium, formally define and evaluate attacker and defender cost functions, construct a Pareto frontier, and assess the robustness of the equilibrium to experimental variance.

### 6.1. Payoff Matrix and Nash Equilibrium

The empirical payoff data are first recorded as a 198 × 8 scenario matrix M, where each cell M[i,j] contains the mean balanced accuracy achieved by attacker classifier *j* against scenario row *i* over 10-fold GroupKFold cross-validation. Because donor rows are duplicated across δ labels, the effective strategic game contains 156 unique defender configurations. Duplicate donor rows are strategically equivalent; removing duplicate rows does not change the minimax value because identical pure strategies provide no additional best-response option.

A corresponding standard deviation matrix $M_{\sigma }$ records the variability across folds for each cell. The complete payoff matrix and the per-configuration top-3 attacker rankings are released with the reproducibility package (see Data Availability Statement).

**Pure-strategy analysis.** Because the defender minimizes balanced accuracy while the attacker maximizes it, the relevant saddle-point conditions for our zero-sum game are:

(1) $$\mathrm{defender}’s\;\mathrm{maximin}:\;v_{D}=\min_{i}\max_{j}M\left[i,j\right],$$

(2) $$\mathrm{attacker}’s\;\mathrm{maximin}:\;v_{A}=\max_{j}\min_{i}M\left[i,j\right].$$

A pure-strategy saddle point exists if and only if $v_{D}=v_{A}$. In our empirical payoff matrix, these two quantities are not equal, so no pure-strategy Nash equilibrium exists. We therefore compute the mixed-strategy Nash equilibrium using linear programming. The minimax theorem for finite zero-sum games guarantees that the mixed equilibrium value $v^{*}$ satisfies $v_{A}\leq v^{*}\leq v_{D}$.

Although no pure-strategy NE exists for the full game, the structure of the matrix has a striking property: if the defender’s strategy space is restricted to synthetic methods only (the 144 fixed/exponential/uniform configurations plus 6 no-defense baselines), for every synthetic configuration, the best-responding attacker exceeds 72% balanced accuracy. In other words, no synthetic-only defense can hold the attacker below ≈72% accuracy in the worst case. This indicates that, within the evaluated timing/size feature set and tabular classifier set, synthetic injection methods provide little protection against a best-response attacker across the tested timing distributions, window sizes, and δ values.

**Mixed-strategy analysis.** When the full effective strategy space is considered, including the six unique donor configurations, the mixed-strategy Nash equilibrium is computed via linear programming. All 48 donor-labeled rows are retained in the released payoff data for reporting consistency, but the donor rows that differ only in δ are duplicate strategies because donor injection ignores δ.

For the attacker, the LP maximizes the game value *v* subject to:

(3) $$\begin{array}{cc} \mathrm{maximize}\; & v\\ \mathrm{subject}\;\mathrm{to}\; & \sum_{j}p_{j}\cdot M\left[i,j\right]\geq v\;\forall i\in S^{D},\\ \; & \sum_{j}p_{j}=1,\;p_{j}\geq 0, \end{array}$$

where $p_{j}$ is the probability of selecting classifier *j*. Symmetrically, the defender’s LP minimizes *v* subject to:

(4) $$\begin{array}{cc} \mathrm{minimize}\; & v\\ \mathrm{subject}\;\mathrm{to}\; & \sum_{i}q_{i}\cdot M\left[i,j\right]\leq v\;\forall j\in S^{A},\\ \; & \sum_{i}q_{i}=1,\;q_{i}\geq 0. \end{array}$$

Both LPs are solved using the HiGHS solver (via SciPy’s linprog). By the minimax theorem for finite zero-sum games, both LPs yield the same game value $v^{*}=0.2466$. The equilibrium strategies are summarized in Table 14.

By convention, donor configurations are labeled with δ=5.0 in equilibrium output and feature-importance analysis; because the donor method ignores δ, this label is arbitrary and has no effect on the results. The defender plays donor-based mimicry exclusively; no synthetic method appears in the equilibrium mix. The only strategic decision is the window size: w=60 s (probability 0.7245) provides the strongest per-window defense because longer windows allow more donor packets to accumulate and dominate the feature statistics, while w=10 s (probability 0.2755) provides a complementary defense with different feature distributions, preventing the attacker from specializing against a single window size.

The attacker abandons all six ensemble and kernel-based classifiers (Random Forest, XGBoost, Extra-Trees, KNN, SVM-RBF, Logistic Regression), retaining only Gaussian NB (probability 0.7283) and MLP (probability 0.2717).

The equilibrium game value of $v^{*}=0.2466$ is close to the 0.25 random-guessing threshold for four device classes. This means that, within the evaluated dataset, feature set, classifier set, and defense configurations, the empirical minimax solution limits the pairing-unaware attacker’s mean balanced accuracy to 24.7%. This should not be interpreted as a universal bound against arbitrary classifiers, raw-sequence models, or 802.11 forensic features outside the evaluated payoff matrix; the pairing-aware relabeling case is quantified in Section 6.7.

### 6.2. Attacker Cost Analysis

The attacker incurs computational costs in training classifiers. We define the attacker’s cost function as the wall-clock training time normalized across classifiers:

(5) $$C^{A}\left(j\right)=t_{j}/\max_{j}t_{j},$$

where $t_{j}$ is the mean training time (in seconds) for classifier *j* averaged across all 198 scenarios and 10 CV folds. Table 15 reports the raw and normalized costs for each classifier.

The equilibrium attacker strategy uses the two cheapest classifiers that retain residual signal under donor injection. The primary equilibrium classifier, Gaussian NB, is the second-cheapest overall (0.002 s training time, normalized cost 0.003), while the secondary classifier, MLP, is moderately expensive (0.285 s, normalized cost 0.444). The most expensive classifiers (Random Forest at 0.641 s, XGBoost at 0.388 s) collapse to near-zero accuracy under donor injection and are excluded from the equilibrium entirely. We note that this cost-accuracy alignment is descriptive rather than prescriptive: the game value $v^{*}=0.2466$ is computed from the payoff matrix M alone, without incorporating attacker cost into the optimization. The equilibrium attacker happens to use cheap classifiers because the same classifiers that are computationally cheap (GNB, MLP) also happen to be the only ones that exploit residual donor-regime signal; the LP did not select them on cost grounds. A cost-weighted formulation that integrates $C^{A}$ into the payoff would be a natural extension.

We use training time rather than inference time as the attacker cost metric because training is the computational bottleneck for the attacker: fitting model parameters requires processing all available labeled data, while classifying a single observation window is negligible for all eight classifiers (<0.001 s per sample). In a realistic attack scenario, the attacker trains once on collected data and then classifies continuously, making the one-time training cost the relevant strategic decision factor.

### 6.3. Defender Cost and Overhead Analysis

The defender’s cost is captured by the mean injection gap, i.e., the average inter-arrival time between consecutive injected packets, which inversely correlates with bandwidth overhead. We define the defender’s cost function as:

(6) $$C^{D}\left(i\right)=\frac{1}{\left|D\right|}\sum_{d\in D}{\bar{a}}_{d}\left(i\right),$$

where ${\bar{a}}_{d}\left(i\right)$ is the mean inter-injection gap for device *d* under defender configuration *i*. A smaller gap implies denser injection and higher bandwidth cost. We use the cross-device mean of ${\bar{a}}_{d}\left(i\right)$ as a coarse scalar cost proxy for the λ-weighted utility analysis below; because the four devices operate at rates spanning four orders of magnitude, this average is not a physically meaningful bandwidth figure (see the per-device overhead discussion later in this section), and the substantive cost comparison between donor and synthetic methods rests on the per-device gaps and the total injection volume rather than on $C^{D}\left(i\right)$ itself.

The defender’s net utility incorporates both privacy and cost:

(7) $$U^{D}\left(i,j\right)=\left(1-M[i,j]\right)-\lambda \cdot C^{D}\left(i\right),$$

where the first term is the privacy gained (1 minus the attacker’s balanced accuracy) and λ is a weighting parameter that controls the cost-privacy tradeoff. We set λ=1.0, treating one unit of privacy (one percentage point of accuracy reduction) as equivalent to one unit of cost (one second of the scalar $C^{D}$ proxy). Because donor configurations achieve privacy values of 0.55–0.74 at approximately 100% per-device overhead while synthetic methods achieve privacy below 0.15 at any overhead level, the privacy advantage of donor methods (∼0.5 on average) far exceeds their cost. Evaluated with the scalar $C^{D}\left(i\right)$ proxy defined above, the equilibrium defender strategy remains donor-based for all λ≤5.0; for substantially larger λ values, the analysis would shift toward sparser configurations. This conclusion does not depend on the proxy: as the per-device and total-volume figures below show, every synthetic configuration is dominated by a donor configuration on privacy at comparable or lower bandwidth cost, so no reasonable cost weighting would prefer a synthetic configuration. The attacker’s net utility is defined symmetrically:

(8) $$U^{A}\left(i,j\right)=M\left[i,j\right]-C^{A}\left(j\right).$$

The donor method’s overhead is determined by the paired device’s traffic volume, not by the δ parameter. For each device, the defender injects a complete replica of the paired donor’s traffic stream, resulting in approximately 100% overhead. However, the absolute overhead varies substantially across device pairs: for the idle pair (Bulb↔Plug), the overhead is 509 additional packets over the entire 30-minute capture; for the active pair (Camera↔Doorbell), it is approximately 850,000 packets. The mean injection gap for donor-based mimicry ranges from approximately 0.0004 s to 0.260 s across the four devices, since each device receives cover traffic at its paired donor’s natural rate (e.g., Doorbell’s stream is injected at Camera’s rate, and Bulb’s stream at Plug’s rate). The aggregate overhead across all four devices is 852,855 injected packets over approximately 1800 s of capture, yielding a combined injection rate of approximately 474 packets per second. The “∼100% overhead” framing throughout this paper reflects per-device traffic doubling. Aggregate network-level overhead is dominated by the active pair: Camera and Doorbell together account for ∼99.9% of all injected packets, while Bulb and Plug together contribute only ∼0.1%. From the perspective of total wireless airtime, the donor defense is therefore essentially an active-device defense with a free idle-device bonus, since the bandwidth cost of protecting Bulb and Plug (∼0.3 packets/sec) is negligible compared to the cost of protecting Camera and Doorbell.

By contrast, synthetic methods at matched overhead (δ=0.01, ∼700,000 injected packets, approximately the same as donor) achieve only 93.9% attacker accuracy, and at 83× higher overhead (δ=0.0001, ∼71 million packets), attacker accuracy increases to 95.2%. The defender’s cost-effectiveness overwhelmingly favors donor-based mimicry.

### 6.4. Pareto Frontier

**Figure 11:**
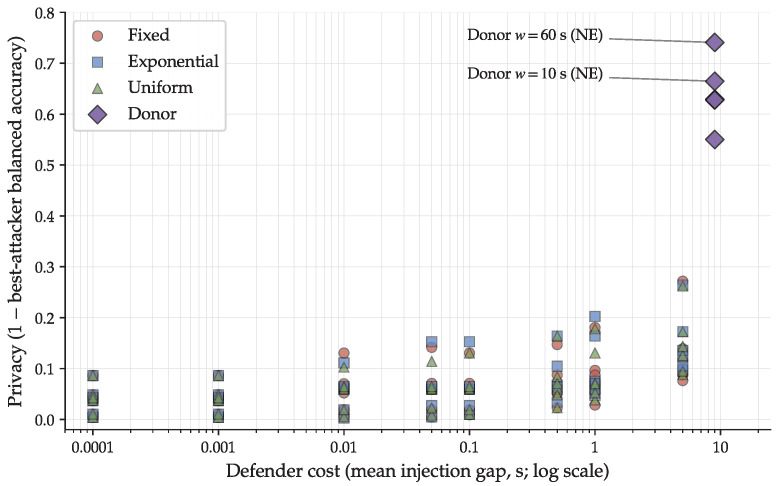
Pareto frontier of privacy versus defender cost. Privacy is defined as $1-\max_{j}M\left[i,j\right]$, where M[i,j] is the attacker’s balanced accuracy against defender configuration *i* using classifier *j*. Defender cost is measured as the mean injection gap (log scale); smaller gaps imply denser injection. Synthetic methods cluster in the low-privacy region across the cost range, while donor methods occupy a distinct high-privacy region. No synthetic configuration is Pareto-optimal.

To visualize the tradeoff between privacy and defender cost, we construct a Pareto frontier plotting privacy (defined as $1-\max_{j}M\left[i,j\right]$, the worst-case privacy for each defender configuration *i*) against defender cost $C^{D}\left(i\right)$. A defender configuration is Pareto-optimal if no other configuration achieves both higher privacy and lower cost. Figure 11 displays this frontier.

The frontier reveals a stark separation. Synthetic methods cluster in the low-privacy region (privacy <0.15) across the entire cost spectrum, from extremely high overhead (δ=0.0001, mean gap ≈0.0001 s) to minimal overhead (δ=5.0, mean gap ≈5.0 s). All three synthetic timing distributions produce nearly identical Pareto points, confirming that the distributional form is irrelevant when the timing is artificial.

Donor methods occupy a distinct cluster in the high-privacy region (privacy 0.55–0.74) at approximately 100% per-device bandwidth overhead. Because the donor method ignores δ, all 48 donor configurations produce similar privacy-cost points. The Pareto-optimal configurations are donor w=60 s (privacy =0.741) and donor w=10 s (privacy =0.665), corresponding to the two strategies in the Nash equilibrium. No synthetic configuration is Pareto-optimal: for every synthetic configuration, a donor configuration achieves strictly higher privacy at comparable or lower cost.

### 6.5. Network Performance Impact

The ∼100% overhead figure used throughout this paper is relative to each protected device’s own traffic. To assess what this costs the network, we translate the injected volume into absolute load and channel airtime using the measured captures. Each device’s bidirectional 802.11 stream (frame bytes, including MAC headers) was measured directly from the traces: Camera transmits and receives 548.9 MB over 1820 s (2.41 Mbps, 346.7 packets/s, mean frame 870 B), Doorbell 140.3 MB over 1807 s (0.62 Mbps, 121.0 packets/s, mean frame 642 B), while Bulb and Plug together generate under 0.3 kbps. Since donor injection replays a replica of the partner’s stream, the defense adds approximately 3.0 Mbps and 468 packets/s of sustained load in aggregate, of which the active pair accounts for over 99.9%, consistent with the 852,855 injected packets reported above (within 0.4%, attributable to replay cycling).

Relative to channel capacity, this load is modest. It corresponds to approximately 6.7% of the effective MAC throughput of a low-end 802.11n access point (20 MHz, one spatial stream, ∼45 Mbps effective) and under 1% for typical 802.11ac configurations. Because Wi-Fi cost is better expressed as airtime when frames are small, we also estimate added channel airtime, accounting for per-frame protocol overhead (DIFS, average backoff, PHY preamble, and block acknowledgment, ∼180 μs per unaggregated frame, amortizing to ∼20 μs per frame under standard A-MPDU aggregation). Without aggregation, the injected traffic occupies approximately 12.6% of channel airtime at a 72.2 Mbps PHY rate (9.4% at 300 Mbps); with aggregation, which a defending AP controls directly since it generates the frames itself, this drops to approximately 5.1% and 2.0%, respectively. These utilization levels are far below the saturation region in which CSMA/CA contention and queuing meaningfully inflate latency for competing traffic, so the expected impact on coexisting flows is small. The injected schedule also inherits the donor’s natural burst structure rather than adding pathological burstiness, and the entire cost is borne by the mains-powered access point: protected IoT devices transmit nothing additional and incur no energy or latency penalty.

Two caveats bound this analysis. First, these are analytic airtime estimates under stated assumptions rather than testbed throughput and latency measurements; a full-stack evaluation under contending foreground traffic is left to future work. Second, the absolute cost scales with the number of protected high-throughput devices: each additional camera-class pair adds load comparable to the pair’s own traffic, so dense deployments would need to budget airtime across devices, for example by protecting only privacy-sensitive devices or by optimizing pairings under an explicit airtime constraint.

### 6.6. Robustness Analysis

The reliability of the Nash equilibrium depends on the stability of the underlying payoff matrix. Each cell is estimated via 10-fold cross-validation, and robustness is assessed through cross-validation standard deviations and the distribution of payoff values relative to the game value. The cell M[uniformw=1s/δ=0.0001,KNN]=0.994±0.011 is estimated with low cross-validation variance, reflecting the saturation of attacker accuracy in this synthetic regime. Across the 1584 payoff matrix cells, synthetic cells (1200 cells, 75.8% of the matrix) cluster at 0.73–0.99 balanced accuracy, more than 5 per-cell standard deviations above the game value $v^{*}=0.2466$. Donor cells (384 cells, 24.2% of the matrix) cluster at 0.10–0.45 and overlap the equilibrium region. This structural separation, rather than within-cell statistical noise, drives the equilibrium support: the LP selects donor configurations because synthetic cells are uniformly outside the range any optimal defender would choose, not because individual donor cells are precisely equal to $v^{*}$.

The equilibrium defender configurations exhibit moderate cross-validation variance. Donor w=60 s achieves a best-attacker balanced accuracy of 0.259±0.180, and donor w=10 s achieves 0.335±0.158. These relatively large standard deviations reflect the small number of test samples per fold at these window sizes; for example, at w=60 s, each device produces approximately 30 windows, yielding roughly three test samples per class per fold. A 95% prediction interval for per-fold best-attacker accuracy at donor w=60 s is approximately [0.00,0.61] using a normal approximation across 10 folds. This interval reflects small per-fold test sets rather than instability in the underlying defense: the central estimate of 0.259 is consistent with the donor regime as a whole (mean 0.170 across 48 donor configurations, see Table 12) and remains more than half a standard deviation below any synthetic-method cell. The equilibrium structure is therefore robust to plausible per-fold fluctuations, although stochastic classifiers (RF, XGBoost, MLP, Extra-Trees) use a fixed random seed (42). Running 5–10 seeds would provide tighter confidence bounds but would multiply the 1584 classifier–configuration pairs to 7920–15,840 model fits; this extension is left for future work, while the current 10-fold design follows standard practice in ML-based security evaluations [4,5].

### 6.7. Pairing-Aware Attacker Analysis

The results reported so far model a pairing-unaware attacker who treats the four-class identification problem at face value. The stable identity swap documented in Section 5, however, suggests a natural adaptive strategy: an attacker who knows, or infers, that donor pairing is deployed can attempt to invert the swapped predictions. This subsection evaluates that adaptive attacker model quantitatively using the framework itself, requiring no assumptions beyond those already present in the payoff matrix.

**Attacker model and metrics.** We model a pairing-aware attacker who knows the donor deployment and the pairing structure and who post-processes the classifier output by applying the label permutation that maximizes balanced accuracy. We refer to this as *oracle relabeling*: among all 4!=24 permutations of the predicted labels, the attacker selects the one yielding the highest balanced accuracy. This is an upper bound on relabeling attacks, but a practically achievable one, since orienting a stable pairwise swap requires only minimal side information, such as a small number of labeled observation windows or knowledge of which physical device is which. We additionally report the *pair-membership* balanced accuracy, obtained by collapsing the four classes into the two behavioral superclasses {Bulb, Plug} and {Camera, Doorbell}, and the *within-pair* balanced accuracy under relabeling, which measures how well the attacker separates the two members of each pair. All three metrics are computed by post-processing the fold-pooled confusion matrices of the existing evaluation; pooling across folds introduces small differences relative to the fold-mean values reported elsewhere (e.g., the fold-pooled best-attacker accuracy at donor w=60 s is 0.264 versus the fold-mean 0.259).

Table 16 reports the results for the six unique donor configurations.

Three findings emerge. First, oracle relabeling restores near-baseline identification accuracy: the pairing-aware attacker achieves 0.852–0.975 balanced accuracy across all donor configurations, compared with the 0.943 baseline maximum. The configuration that is strongest against the pairing-unaware attacker (w=60 s, where Random Forest falls to 0.000) is the most vulnerable to relabeling (0.975), precisely because its identity swap is the most stable. In every donor cell except MLP at w=30 s and w=120 s, the optimal permutation is the full pair swap Bulb↔Plug, Camera↔Doorbell.

Second, the classifier ranking inverts a second time. The ensemble classifiers that collapse to near-zero accuracy under the pairing-unaware evaluation become the strongest pairing-aware attackers, because their errors are systematic and therefore invertible. Conversely, Gaussian NB and MLP, the only classifiers in the pairing-unaware equilibrium support, are the weakest attackers under relabeling (0.460–0.780): their confusion is genuinely mixed rather than systematically swapped, so no permutation recovers it.

Third, recomputing the Nash equilibrium with every payoff cell replaced by its oracle-relabeling value raises the game value from 0.2525 to 0.8010 on the fold-pooled matrices. The equilibrium attacker shifts back to ensemble and kernel classifiers (Extra-Trees, 58.4%; SVM-RBF, 41.6%), and the equilibrium defender abandons donor configurations entirely, retreating to synthetic configurations at w=1 s whose residual advantage stems from the intrinsic statistical difficulty of one-second windows rather than from the injection itself. No defense configuration in the evaluated strategy space holds the pairing-aware attacker below 0.826 balanced accuracy.

The practical interpretation is that donor mimicry provides strong protection against pairing-unaware attackers and pair-level anonymity against pairing-aware attackers: even under oracle relabeling, the attacker learns which behavioral pair a device belongs to (pair-membership accuracy 0.972–1.000, information largely available from aggregate traffic volume alone), and the defense’s four-class protection is conditional on the attacker not knowing or not being able to orient the pairing. This makes pairing secrecy an explicit deployment requirement and motivates countermeasures that destabilize the swap, such as periodically rotating the pairing assignment, randomizing the injection orientation within a pair, and combining donor injection with timestamp perturbation, which we discuss as future work in Section 7.5.

## 7. Discussion

### 7.1. Why Donor Mimicry Works and Synthetic Methods Fail

The central finding of this work is the stark asymmetry between donor-based mimicry and synthetic injection methods. At matched injection volume (∼850,000 packets, ∼100% overhead), donor reduces the best attacker’s balanced accuracy to 33.5%, while fixed, exponential, and uniform injection all achieve 93.9%. At 83× higher volume (70.8 million packets), synthetic methods still achieve 95.2% attacker accuracy. This section explains the mechanism behind this asymmetry.

**Figure 12:**
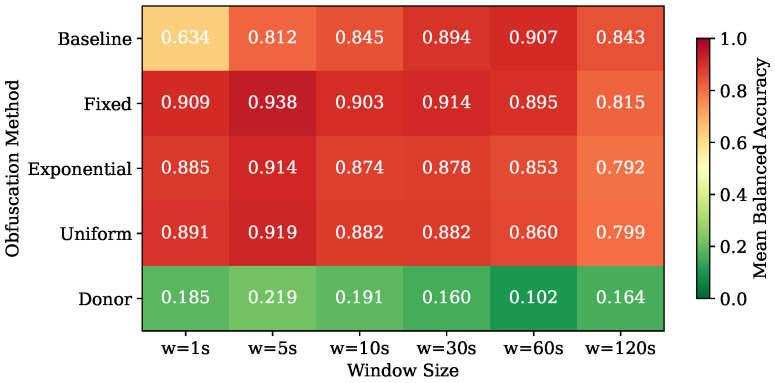
Aggregated attacker balanced accuracy by the obfuscation method and window size, averaged across all classifiers and δ values. The donor method, bottom row, achieves dramatically lower accuracy than all synthetic methods and baseline across every window size, confirming that behavioral mimicry is the decisive factor regardless of observation duration.

The explanation lies in the interaction between injected traffic characteristics and the feature extraction pipeline. All four methods inject packets with sizes drawn from the donor device’s real packet size distribution. The difference is exclusively in timing. Synthetic methods inject at rates controlled by δ: constant for fixed, memoryless random for exponential, and bounded random for uniform. These artificial timing patterns introduce detectable regularities into the inter-arrival time features. A fixed injection at δ=0.01 s produces a spike at 0.01 s in the IAT distribution that no real device exhibits. Exponential injection at mean δ produces a memoryless IAT distribution that similarly has no real-device counterpart. The classifiers learn to identify and exploit these artificial timing signatures, effectively filtering out the injected packets and recovering the original device’s statistical profile. Figure 12 summarizes this contrast, showing the mean attacker accuracy aggregated across all classifiers and δ values for each method and window size combination.

The donor method avoids this pitfall by injecting the paired device’s real inter-arrival pattern, so the combined traffic approximates a realistic mixture of both devices under the evaluated windowed timing, size, and throughput features.

The confusion matrices show that, under donor injection, several pairing-unaware classifiers map devices to their paired partners with near-certainty, for example Bulb→Plug 100% and Camera→Doorbell 98%. This is best interpreted as a systematic disruption of the original feature-label relationship. It is stronger than random confusion for the pairing-unaware attacker, but it also creates a potential relabeling vulnerability if an attacker knows the donor pairing and observes that the mapping is stable.

At the feature level, the mechanism appears as convergence: under donor at the equilibrium-relevant w=60 s, RF, GNB, and MLP all collapse to balanced size/timing importance distributions with no feature exceeding 0.12 (Table 9 and Table 10), matching RF’s near-zero accuracy and the residual capability of GNB (0.259) and MLP (0.213).

### 7.2. The Attacker’s Equilibrium Response

The Nash equilibrium reveals that a rational pairing-unaware attacker facing donor-based mimicry should abandon ensemble classifiers entirely. Random Forest, XGBoost, and Extra-Trees, which are the strongest classifiers under baseline conditions (94.3%, 93.6%, and 94.3% balanced accuracy, respectively), achieve near-zero accuracy under donor injection because they rely on feature interactions (e.g., correlations between size variance and IAT variance) that the donor method disrupts completely. These classifiers are designed to exploit correlated feature structures, and when the injection merges two devices’ feature distributions, those correlations become noise.

The equilibrium attacker instead selects Gaussian NB (72.8% probability) and MLP (27.2%). This combination reflects two distinct strategies for exploiting residual signal in donor-obfuscated traffic. GNB treats each feature independently, so even partial survival of one feature’s discriminative power is sufficient. MLP, with its (128, 64) hidden layer architecture, can learn nonlinear combinations of timing features that GNB’s independence assumption prevents. However, even the best attacker (MLP at donor w=30 s) achieves only 45.0% balanced accuracy, which is well below the baseline and insufficient for reliable device identification.

This finding has practical implications for both sides. For the defender, the existence of a residual vulnerability through timing features suggests that future defenses could be strengthened by combining donor injection with timestamp perturbation, adding controlled jitter to the original device’s real packet timestamps. For the attacker, the cheapest adaptive avenue is the pairing-aware relabeling quantified in Section 6.7; beyond it, more complex classifiers (e.g., deep recurrent networks operating on raw packet sequences) could exploit sequential dependencies that windowed statistical features cannot capture.

The per-configuration top-three attacker rankings in the released payoff data confirm that Gaussian NB and MLP are the rank-1 attackers against donor configurations and that ensemble methods are the rank-1 attackers against synthetic configurations.

### 7.3. Practical Deployment Considerations

The proposed defense is designed as an AP-level mechanism in which the access point generates locally absorbed dummy 802.11 frames associated with paired devices. This deployment model avoids IoT firmware modification and uses the existing AP as the defense point.

The primary deployment cost is bandwidth overhead. For the idle pair (Bulb/Plug), donor mimicry adds negligible absolute traffic: 509 packets over 30 min. For the active pair (Camera/Doorbell), the added load is approximately 3.0 Mbps of sustained traffic; Section 6.5 quantifies this as a few percent of channel airtime on commodity access points, small enough to leave coexisting traffic unaffected, though dense deployments with many high-throughput devices would need to budget airtime explicitly.

De-obfuscation is inherent to the architecture because the AP generates the dummy frames and can keep them separate from real device traffic in its forwarding pipeline. Real traffic follows the normal routing path to the internet gateway, whereas dummy traffic is transmitted only over the wireless medium. This is analogous to existing AP-level traffic management functions such as QoS shaping, broadcast filtering, and management frame processing.

Device pairing is a one-time configuration step. In production, the AP could discover device behavioral profiles during an initial observation period and compute pairings that maximize confusion while controlling bandwidth overhead. Networks with unpaired devices may require fallback strategies, such as hybrid donor-plus-timestamp perturbation. The full evaluation (15,840 model fits) required approximately 12 h on an HPC cluster.

### 7.4. Donor Availability and Selection

The donor method as evaluated assumes that each protected device has a behaviorally similar partner, which held naturally in our testbed. Realistic smart homes will not always offer such pairs, so we outline a donor-selection strategy for that case, ordered from the least to the most structural change.

**Similarity-based matching.** Donor selection can be formalized as a matching problem on behavioral profiles. The defender, who observes all local traffic at the AP, computes each device’s profile using the same windowed features available to the attacker and pairs devices to minimize a distributional distance between profiles (for example, the energy or Wasserstein distance over the per-window feature distributions). Our Bulb↔Plug and Camera↔Doorbell pairs are the outcome such matching would produce on this testbed. When only an imperfect partner is available, the defender pairs with the nearest device and accepts weaker merging of the two distributions; we expect effectiveness to degrade gracefully with behavioral distance, since the volume-ratio results of Section 5 indicate that behavioral realism rather than injected volume drives obfuscation, but quantifying this degradation requires multi-device captures and is part of the future work discussed below.

**Virtual donors from recorded traces.** More fundamentally, the donor is a traffic pattern, not a physical device: injection is generated entirely by the AP, so the replayed schedule need not originate from a co-located device. A device without a suitable partner can be assigned a virtual donor drawn from a library of authentic recorded traces (from prior deployments, public IoT traffic corpora, or vendor-provided profiles) selected by the same similarity criterion. This preserves the property that separates donor mimicry from the synthetic baselines, namely that the injected traffic follows genuine device behavior, while removing the dependence on the local device population. The residual risks are that a mismatched virtual donor weakens the merge, and that an attacker with knowledge of the home’s inventory might flag traffic resembling a device that is not present; both argue for selecting virtual donors of plausible, common device types.

**Group donors.** For odd device counts or clusters of similar devices, pairing generalizes to donor groups of size k>2, in which each member receives replicas drawn from the other members. This extends the pairwise identity swap toward *k*-member confusion within the group, at proportionally higher overhead, and connects to the multi-device game formulations noted under Future Work.

**Hybrid fallback.** A device for which no acceptable real or virtual donor exists can fall back to donor-plus-timestamp-perturbation or to idle-period traffic inflation, trading some of the realism advantage for independence from donor availability.

Donor selection also interacts with cost: the injected volume equals the donor’s volume, so assigning a high-rate donor to a low-rate device imposes the donor’s bandwidth footprint on that stream. In dense deployments, the matching objective should therefore include the airtime budget of Section 6.5 alongside behavioral similarity. Finally, when several acceptable donors exist for a device, rotating among them over time destabilizes the prediction inversion exploited by the pairing-aware attacker of Section 6.7, so donor diversity serves both availability and robustness.

### 7.5. Limitations

This work has several limitations that should be considered when interpreting the results.

**Small device population and single environment.** Our evaluation uses four IoT devices captured in one residential Wi-Fi environment. The scale is a deliberate worst-case choice as well as a constraint: results in the small anonymity-set regime are conservative with respect to population size, since masking is hardest when the attacker must separate only a few candidates. In larger smart homes, the diversity of available donor traffic may increase the number of viable pairings and enlarge anonymity sets, although this scalability hypothesis requires validation on multi-device captures. Scaling donor pairing to production populations of 10–50 devices also introduces combinatorial structure, with possible pairings growing as (N−1)!! and pairing quality depending on the joint behavioral profile of all devices. Separately, all traffic was captured behind a single router in one residential setting; network conditions such as interference, channel utilization, and distance affect packet timing and could alter injection effectiveness, so evaluation across environments and AP models is needed to establish external validity. Both extensions are discussed under Future Work.

**Pairing-aware attackers.**Section 6.7 shows that an oracle-relabeling attacker who knows the pairing recovers 0.852–0.975 balanced accuracy against donor configurations, so the four-class protection is conditional on pairing secrecy. The oracle-relabeling model is an upper bound within the evaluated pipeline; it does not cover attackers who retrain with pairing-aware objectives, adapt online to pairing rotation, or exploit features outside the evaluated set. Evaluating orientation-randomization and pairing-rotation countermeasures against such attackers remains open.

**Windowed statistical feature set.** Our feature set extracts windowed statistics from packet sizes, inter-arrival times, and throughput. It does not include raw packet sequences, 802.11 direction/subtype fields, sequence-number behavior, retransmission patterns, channel-state information, or physical-layer fingerprints. Advanced attackers could employ raw packet sequence models (e.g., 1D-CNNs or LSTMs operating directly on timestamp/size sequences) that exploit sequential dependencies not captured by windowed statistical features. Evaluating the defense against such deep sequential models is left for future work.

**Traffic volume anomaly detection.** A sophisticated attacker could detect the deployment of the defense by observing abrupt traffic-volume changes when injection begins. This does not reveal the donor pairing, but it may signal that cover traffic is active. Future work should evaluate injection-onset concealment strategies, such as pre-activation or gradual ramp-up.

### 7.6. Future Work

The findings and limitations of this work suggest several directions for future research.

**Timestamp perturbation.** Future work should evaluate donor injection combined with controlled jitter or delay of real packet transmission times. This would target residual timing features without additional bandwidth cost, but would introduce a latency–privacy tradeoff that should be incorporated into the game-theoretic framework.

**Dummy traffic inflation for idle periods.** Future work should evaluate whether sustained donor-like cover traffic during idle periods can further reduce the residual fingerprint of low-activity devices such as Bulb and Plug, while accounting for the added bandwidth cost.

**Larger heterogeneous device populations.** Extending the framework to networks with 10–50 devices requires automated pairing algorithms that maximize confusion across behaviorally similar devices while minimizing bandwidth overhead. Future work should quantify what fraction of realistic IoT populations admits viable donor pairing, how residual unpaired devices can be protected, and whether multi-device game formulations can jointly optimize pairings and injection strategies. The donor-selection strategies outlined in Section 7.4 should be evaluated on such captures, including the degradation of obfuscation effectiveness with donor-similarity distance.

**Deep learning attackers.** Future work should evaluate whether donor mimicry remains effective against raw-sequence classifiers, including 1D-CNN, LSTM, and Transformer-based models.

**Adaptive and online attackers.** Modeling the attacker as an adaptive agent who can retrain or update their classifier in response to observed defense changes would bring the game-theoretic framework closer to a repeated game or Stackelberg formulation, where the defender commits to a strategy and the attacker best-responds with full knowledge of the defense.

## 8. Conclusions

This paper presented a finite-game framework for evaluating cover traffic injection defenses against Wi-Fi MAC-layer IoT device fingerprinting. The framework compares synthetic injection methods with donor-based mimicry against a strategic attacker selecting among representative tabular classifiers.

The results support four principal findings within the evaluated dataset, feature set, and classifier set. First, fixed, exponential, and uniform synthetic injection provide little protection against the best-response tabular attacker, even at high bandwidth overhead. Second, donor-based mimicry substantially reduces pairing-unaware attacker performance by merging the protected stream with a behaviorally realistic paired-device trace. Third, the finite-game analysis yields a mixed-strategy Nash equilibrium close to the four-class random-guessing baseline, with the defender using donor configurations and the attacker randomizing primarily between Gaussian NB and MLP. Fourth, a pairing-aware relabeling analysis shows that this protection is conditional on pairing secrecy: an attacker who can orient the donor-induced identity swap recovers 0.852–0.975 balanced accuracy, and the corresponding pairing-aware game value rises to 0.801.

The framework provides an empirical minimax bound within the evaluated strategy space: under optimal randomized play across the two equilibrium donor configurations, the donor defense limits the pairing-unaware attacker’s mean balanced accuracy to 24.7%. These results establish behaviorally realistic donor traffic as an effective defense principle against pairing-unaware MAC-layer fingerprinting, with commodity-AP implementation, pairing-aware countermeasures, larger device populations, and raw-sequence attackers remaining as future work.

## Figures and Tables

**Table 1:** Comparison of related traffic obfuscation defenses against IoT device fingerprinting.

Work	Year	Defense Type	Layer	Features	Classifiers	Game Theory	Volume Analysis
[1]	2022	Paired-device shaping	MAC	2	3	No	No
[16]	2023	Random segmentation	MAC	Size-based	4	No	No
[10]	2022	Eight padding methods	MAC	Full size dist.	1 (RF)	No	No
[17]	2019	Stochastic padding	Network	Traffic rates	Theoretical	No	Partial
[18]	2022	Dynamic dummy traffic	Network	Timing	2	No	No
[19]	2022	DP-based shaping	Network	Mixed	3	No	No
[11]	2025	GAN-based injection	Network	Mixed	3	No	No
[24]	2024	Moving target defense	System	RF fingerprint	RL-based	Partial (RL)	No
**This work**	**2026**	**Donor mimicry + 3 synthetic**	**MAC**	**18 features**	**8 (all families)**	**Full NE (198 × 8)**	**Yes**

**Table 2:** Device characteristics.

Device	MAC Address	Packets	Duration	Median IAT	Idle %	Donor
Bulb	48:e1:e9:1a:22:6c	459	1784 s	0.115 s	89.1%	Plug
Plug	d8:47:32:c2:24:be	50	1605 s	0.260 s	98.7%	Bulb
Doorbell	9c:8e:cd:27:1f:3b	218,551	1807 s	0.003 s	0.1%	Camera
Camera	2c:aa:8e:8f:74:2d	630,907	1820 s	0.0004 s	17.5%	Doorbell

**Table 3:** Feature definitions and categories for the 18 statistical features extracted per observation window.

Feature	Mathematical Definition	Unit	Category
total_packets	n=|P|, count of packets in window	count	size
total_bytes	$\sum s_{i}$, sum of packet lengths	bytes	size
unique_sizes	$\left|\{s_{i}\}\right|$, count of distinct packet sizes	count	size
avg_pkt_size	$\left(1/n\right)\sum s_{i}$, mean packet length	bytes	size
std_pkt_size	$\sqrt{\left(1/n\right)\sum {\left(s_{i}-\mu \right)}^{2}}$	bytes	size
max_pkt_size	$\max(s_{i})$, maximum packet length	bytes	size
mode_pkt_size	$\arg\max_{v}\left|\left\{i:s_{i}=v\right\}\right|$, most frequent size	bytes	size
var_pkt_size	$\left(1/n\right)\sum {\left(s_{i}-\mu \right)}^{2}$, variance of sizes	bytes^2^	size
var_size_freq	Variance of size-frequency counts	count^2^	size
mean_iat	Mean inter-arrival time	seconds	timing
median_iat	Median IAT	seconds	timing
var_iat	Variance of IATs	s^2^	timing
std_iat	Standard deviation of IATs	seconds	timing
skew_iat	Skewness of IATs	—	timing
kurt_iat	Excess kurtosis of IATs	—	timing
min_iat	Minimum IAT	seconds	timing
max_iat	Maximum IAT	seconds	timing
bytes_per_sec	total_bytes / window duration	bytes/s	rate

**Table 4:** Zero-window prevalence under baseline (no obfuscation) conditions. Values shown as zero-windows/total-windows (percentage).

Device	w=1 s	w=10 s	w=60 s	w=120 s
Bulb	1591/1785 (89.1%)	57/180 (31.7%)	1/31 (3.2%)	1/16 (6.2%)
Plug	1585/1606 (98.7%)	144/162 (88.9%)	14/28 (50.0%)	2/15 (13.3%)
Doorbell	3/1808 (0.2%)	1/182 (0.5%)	1/32 (3.1%)	1/17 (5.9%)
Camera	319/1821 (17.5%)	1/183 (0.5%)	1/32 (3.1%)	1/17 (5.9%)

**Table 5:** Hyperparameter specifications and attacker rationale per classifier.

Model	Family	Hyperparameters	Attacker Rationale
Random Forest	Classical Ensemble	n_estimators=200, max_depth=None, min_samples_leaf=2	Widely used baseline ensemble; robust to overfitting.
XGBoost	Gradient Boosting	n_estimators=200, max_depth=6, lr=0.1, subsample=0.8	State-of-the-art gradient boosting; top performer on tabular data.
KNN	Instance-Based	k=5, weights=distance, metric=minkowski	Non-parametric; captures local decision boundaries.
SVM-RBF	Kernel Method	kernel=rbf, C=10.0, gamma=scale	Powerful nonlinear classifier via RBF kernel.
Logistic Regression	Linear Model	C=1.0, solver=lbfgs, max_iter=1000	Linear baseline; tests linear separability.
MLP	Neural Network	layers=(128,64), relu, early_stopping	Feedforward NN; tests deep nonlinear representations.
Gaussian NB	Probabilistic	(no hyperparameters)	Naive Bayes; extremely fast baseline.
Extra-Trees	Randomized Ensemble	n_estimators=200, max_depth=None, min_samples_leaf=2	More randomized than RF; often lower variance.

**Table 6:** Class balance: number of observation windows per device at representative window sizes under baseline conditions.

Window	Bulb	Camera	Doorbell	Plug
1 s	25.4% (1785)	25.9% (1821)	25.8% (1808)	22.9% (1606)
10 s	25.5% (180)	25.9% (183)	25.7% (182)	22.9% (162)
60 s	25.2% (31)	26.0% (32)	26.0% (32)	22.8% (28)
120 s	24.6% (16)	26.2% (17)	26.2% (17)	23.1% (15)

**Table 7:** Baseline balanced accuracy (mean ± std over 10-fold CV) for all classifiers across observation window sizes. Bold indicates the highest accuracy per column.

Classifier	w=1 s	w=5 s	w=10 s	w=30 s	w=60 s	w=120 s
Random Forest	0.605±0.163	0.847±0.123	0.889±0.074	0.933±0.101	0.943±0.106	0.882±0.171
XGBoost	0.607±0.164	0.848±0.119	0.890±0.071	0.936±0.102	0.936±0.104	0.915±0.118
KNN	0.731±0.234	0.758±0.226	0.751±0.225	0.888±0.084	0.922±0.103	0.774±0.190
SVM-RBF	0.596±0.165	0.818±0.128	0.869±0.073	0.894±0.098	0.886±0.131	0.888±0.152
Logistic Reg.	0.598±0.165	0.817±0.127	0.865±0.075	0.853±0.118	0.883±0.163	0.872±0.181
MLP	0.600±0.160	0.766±0.153	0.784±0.179	0.800±0.156	0.819±0.188	0.653±0.257
Gaussian NB	0.727±0.227	0.792±0.137	0.826±0.119	0.902±0.131	0.926±0.131	0.877±0.170
Extra-Trees	0.607±0.163	0.850±0.120	0.889±0.075	0.943±0.104	0.943±0.106	0.882±0.171

**Table 8:** Controlled volume comparison (w=10 s).

Configuration	Injected Packets	Ratio	Best Attacker Acc.
Donor (any δ)	852,855	1.0×	0.335
Fixed δ=0.01 (matched)	707,000	0.8×	0.939
Exponential δ=0.01 (matched)	743,953	0.9×	0.939
Uniform δ=0.01 (matched)	706,614	0.8×	0.939
Fixed δ=0.001 (8× more)	7,070,510	8.3×	0.952
Fixed δ=0.0001 (83× more)	70,772,621	83×	0.952

**Table 9:** Feature importance at observation window w=30 s for the three equilibrium-relevant classifiers (RF, GNB, MLP), under baseline (BL) and donor (DN) conditions. RF uses impurity-based Gini importance; GNB and MLP use permutation importance on test folds (10 repeats per fold, averaged across 10 GroupKFold folds, scored by balanced accuracy). Each column is normalized to sum to 1.0 across the 18 features.

Feature	Category	BL_RF	DN_RF	BL_GNB	DN_GNB	BL_MLP	DN_MLP
total_packets	Size	0.0949	0.0582	0.0697	0.0316	0.0029	0.0287
total_bytes	Size	0.0814	0.0727	0.0742	0.0409	0	0.0211
unique_sizes	Size	0.0726	0.0590	0.0616	0.0323	0.0029	0.0236
avg_pkt_size	Size	0.0137	0.0810	0.0422	0.0700	0.0039	0.0496
std_pkt_size	Size	0.1052	0.0786	0.0616	0.0367	0.0828	0.0558
max_pkt_size	Size	0.0729	0.0244	0.0606	0.0355	0.0955	0.0306
mode_pkt_size	Size	0.0351	0.0182	0.0564	0.0438	0.0292	0.1541
var_pkt_size	Size	0.1078	0.0872	0.0616	0.0552	0.0799	0.0556
var_size_freq	Size	0.0692	0.0797	0.1054	0.0604	0	0.0333
mean_iat	Timing	0.0418	0.0679	0.0609	0.0917	0.1846	0.1516
median_iat	Timing	0.0380	0.0266	0.0123	0.0389	0.0341	0.0554
var_iat	Timing	0.0269	0.0445	0.0761	0.0624	0.0268	0.0636
std_iat	Timing	0.0294	0.0550	0.0604	0.0955	0.0166	0.1274
skew_iat	Timing	0.0511	0.0290	0.0126	0.0507	0.0429	0.0040
kurt_iat	Timing	0.0312	0.0295	0.0586	0.0571	0.0205	0.0020
min_iat	Timing	0.0114	0.0624	0.0214	0.0643	0.0044	0.0050
max_iat	Timing	0.0259	0.0414	0.0302	0.0922	0.3731	0.0938
bytes_per_sec	Rate	0.0917	0.0849	0.0742	0.0409	0	0.0449
Size (sum)	—	0.6528	0.5590	0.5932	0.4064	0.2971	0.4524
Timing (sum)	—	0.2555	0.3562	0.3326	0.5527	0.7029	0.5027
Rate (sum)	—	0.0917	0.0849	0.0742	0.0409	0	0.0449

**Table 10:** Feature importance at observation window w=60 s, under baseline (BL) and donor (DN) conditions. Columns and normalization as in Table 9. Within-window method effects are reported separately in Table 11.

Feature	Category	BL_RF	DN_RF	BL_GNB	DN_GNB	BL_MLP	DN_MLP
total_packets	Size	0.0700	0.0564	0.0740	0.0123	0	0.0828
total_bytes	Size	0.0708	0.0742	0.0812	0.0102	0	0.0438
unique_sizes	Size	0.0797	0.0389	0.0551	0.0596	0.0138	0.0540
avg_pkt_size	Size	0.0144	0.0778	0.0399	0.1190	0	0.0590
std_pkt_size	Size	0.0939	0.0737	0.0551	0.1190	0.1451	0.0480
max_pkt_size	Size	0.0782	0.0266	0.0551	0.0964	0.1919	0.0301
mode_pkt_size	Size	0.0136	0.0090	0.0524	0.0327	0.0133	0.0581
var_pkt_size	Size	0.1024	0.0801	0.0551	0.0273	0.1478	0.0583
var_size_freq	Size	0.0448	0.0769	0.0971	0.0155	0	0.0466
mean_iat	Timing	0.0564	0.0613	0.0524	0.0857	0	0.0673
median_iat	Timing	0.0312	0.0417	0.0091	0.0482	0.0083	0.0560
var_iat	Timing	0.0564	0.0574	0.0927	0.0675	0.0557	0.0696
std_iat	Timing	0.0557	0.0694	0.0576	0.0985	0.0041	0.0611
skew_iat	Timing	0.0707	0.0291	0.0090	0.0289	0.0908	0.0811
kurt_iat	Timing	0.0330	0.0329	0.0697	0.0375	0.0248	0.0109
min_iat	Timing	0.0055	0.0463	0.0220	0.0653	0.0309	0.0705
max_iat	Timing	0.0588	0.0639	0.0413	0.0664	0.2599	0.0393
bytes_per_sec	Rate	0.0644	0.0847	0.0812	0.0102	0.0138	0.0635
Size (sum)	—	0.5679	0.5135	0.5650	0.4919	0.5118	0.4806
Timing (sum)	—	0.3676	0.4018	0.3538	0.4979	0.4744	0.4559
Rate (sum)	—	0.0644	0.0847	0.0812	0.0102	0.0138	0.0635

**Table 11:** Within-window method effects on category-level feature importance. Each row reports the donor-minus-baseline change in the Size, Timing, and Rate importance aggregates at a fixed window size, computed from the columns of Table 9 and Table 10. Positive values indicate importance gained under donor injection; negative values indicate importance lost. RF and GNB lose size importance and gain timing importance at both windows; MLP shows a timing-to-size shift at w=30 s and a near-null shift at w=60 s.

Classifier	Window	Δ Size	Δ Timing	Δ Rate
RF	w=30 s	−0.0938	0.1007	−0.0068
RF	w=60 s	−0.0544	0.0342	0.0203
GNB	w=30 s	−0.1868	0.2201	−0.0333
GNB	w=60 s	−0.0731	0.1441	−0.0710
MLP	w=30 s	0.1553	−0.2002	0.0449
MLP	w=60 s	−0.0312	−0.0185	0.0497

**Table 12:** Marginal decomposition of attacker balanced accuracy and macro-F1 by experimental factor. Each row averages across all other factors. The dispersion column is the cell-to-cell standard deviation across the marginal, not the per-cell cross-validation standard deviation.

Factor	Configuration	Bal. Accuracy	Macro-F1	Number of Cells
Method	Baseline	0.822±0.105	0.466±0.074	48
	Fixed	0.895±0.080	0.530±0.041	384
	Exponential	0.866±0.103	0.515±0.056	384
	Uniform	0.872±0.100	0.519±0.053	384
	**Donor**	0.170±0.102	0.089±0.054	384
Window size	1 s	0.715±0.326	0.392±0.188	264
	5 s	0.750±0.311	0.417±0.173	264
	10 s	0.716±0.305	0.427±0.185	264
	30 s	0.714±0.326	0.427±0.196	264
	60 s	0.684±0.339	0.404±0.205	264
	120 s	0.648±0.287	0.421±0.202	264
Classifier	Random Forest	0.721±0.361	0.428±0.213	198
	XGBoost	0.719±0.361	0.426±0.212	198
	KNN	0.688±0.326	0.409±0.194	198
	SVM-RBF	0.696±0.311	0.406±0.196	198
	Logistic Regression	0.692±0.308	0.407±0.191	198
	MLP	0.661±0.226	0.389±0.140	198
	Gaussian NB	0.736±0.262	0.427±0.165	198
	Extra-Trees	0.723±0.355	0.427±0.210	198
δ (synthetic only)	0.0001 s	0.928±0.076	0.549±0.039	144
	0.001 s	0.929±0.072	0.550±0.037	144
	0.01 s	0.901±0.090	0.532±0.041	144
	0.05 s	0.880±0.100	0.520±0.045	144
	0.1 s	0.875±0.103	0.517±0.045	144
	0.5 s	0.861±0.080	0.514±0.040	144
	1.0 s	0.842±0.087	0.504±0.052	144
	5.0 s	0.807±0.086	0.484±0.064	144

**Table 13:** Method × classifier mean balanced accuracy by obfuscation method and attacker classifier. Each cell reports mean balanced accuracy averaged across all relevant windows and δ values for that method-classifier combination. Baseline uses six cells per classifier, synthetic methods use 48 cells per classifier, and donor uses 48 donor-labeled cells per classifier, corresponding to six unique donor configurations repeated across δ labels. The best attacker per row is shown in bold and the worst attacker per row is italicized.

Method	RF	XGBoost	Extra-Trees	KNN	SVM-RBF	LogReg	GNB	MLP
Baseline	0.850	**0.855**	0.852	0.804	0.825	0.814	0.841	*0.737*
Fixed	**0.927**	0.925	0.922	0.912	0.890	0.888	0.881	*0.818*
Exponential	**0.923**	0.920	0.922	0.837	0.850	0.846	0.877	*0.754*
Uniform	0.924	**0.925**	0.925	0.858	0.861	0.851	0.878	*0.757*
Donor	*0.093*	*0.090*	0.108	0.130	0.168	0.170	0.294	**0.307**

**Table 14:** Nash equilibrium strategies ($v^{*}=0.2466$).

Player	Strategy	Probability	Role
Defender	Donor w=60 s/δ=5.0	0.7245	Primary defense
Defender	Donor w=10 s/δ=5.0	0.2755	Secondary defense
Attacker	Gaussian NB	0.7283	Primary classifier
Attacker	MLP	0.2717	Secondary classifier

**Table 15:** Attacker training costs.

Classifier	Family	Raw Time (s)	$C^{A}\left(j\right)$	In NE?
KNN	Instance-Based	0.001	0.002	No
Gaussian NB	Probabilistic	0.002	0.003	Yes (72.8%)
Logistic Reg.	Linear	0.070	0.110	No
SVM-RBF	Kernel	0.105	0.164	No
MLP	Neural Network	0.285	0.444	Yes (27.2%)
Extra-Trees	Randomized Ens.	0.359	0.559	No
XGBoost	Gradient Boost.	0.388	0.605	No
Random Forest	Classical Ens.	0.641	1.000	No

**Table 16:** Pairing-aware attacker evaluation across the six unique donor configurations. Each column reports the strongest attacker classifier for that metric. Oracle relabeling applies the balanced-accuracy-maximizing label permutation to the classifier output; pair membership is the two-superclass problem {Bulb, Plug} vs. {Camera, Doorbell}; within-pair is the two-class separation inside each pair after relabeling.

Window	Best Pairing-Unaware	Best Oracle Relabeling	Pair Membership	Within-Pair (Idle)	Within-Pair (Active)
1 s	0.365 (GNB)	0.888 (RF)	1.000	0.822	0.999
5 s	0.373 (GNB)	0.852 (SVM-RBF)	0.999	0.824	0.999
10 s	0.335 (MLP)	0.865 (SVM-RBF)	0.997	0.860	0.995
30 s	0.460 (MLP)	0.959 (KNN)	0.996	0.949	1.000
60 s	0.264 (GNB)	0.975 (XGBoost)	0.985	1.000	1.000
120 s	0.401 (MLP)	0.915 (Extra-Trees)	0.972	1.000	0.944

## Data Availability

The original contributions presented in this study are included in the article. The IoT traffic captures were collected in a controlled laboratory environment. The simulation scripts, classification pipeline, game-theoretic analysis code, and the complete 198 × 8 empirical payoff matrix are openly available at https://github.com/m-jeed/wifi-mac-donor-obfuscation (version 1.0.0, accessed on 10 July 2026). The raw packet captures are available from the corresponding author on reasonable request.

## References

[1] Alyami M., Alkhowaiter M., Al Ghanim M., Zou C., Solihin Y. MAC-Layer Traffic Shaping Defense Against WiFi Device Fingerprinting Attacks. Proceedings of the 27th IEEE Symposium on Computers and Communications (ISCC).

[2] Bezawada B., Bachani M., Peterson J., Shirazi H., Ray I. Behavioral Fingerprinting of IoT Devices. Proceedings of the ACM CCS Workshop on Attacks and Solutions in Hardware Security.

[3] Msadek N., Soua R., Engel T. IoT Device Fingerprinting: Machine Learning Based Encrypted Traffic Analysis. Proceedings of the IEEE Wireless Communications and Networking Conference (WCNC).

[4] Skowron M., Janicki A., Mazurczyk W. (2020). Traffic Fingerprinting Attacks on Internet of Things Using Machine Learning. IEEE Access.

[5] Bezawada B., Ray I., Ray I. (2021). Behavioral Fingerprinting of Internet-of-Things Devices. WIREs Data Min. Knowl. Discov..

[6] Mir M.M., Tan W.L., Awrangjeb M., Zia A. A Comparative Analysis of IoT Device Fingerprinting Methods. Proceedings of the 34th International Telecommunication Networks and Applications Conference (ITNAC).

[7] Baena E., Yang H., Koutsonikolas D., Haque I. (2025). A Comprehensive Survey on Smart Home IoT Fingerprinting: From Detection to Prevention and Practical Deployment. arXiv.

[8] Li R., Liu S., Hu H., Ye Q., Feamster N. (2025). WiFinger: Fingerprinting Noisy IoT Event Traffic Using Packet-level Sequence Matching. arXiv.

[9] Gong J., Cai W., Liang S., Guan Z., Wang T., Chang E.C. (2026). WFCAT: Augmenting Website Fingerprinting with Channel-wise Attention on Timing Features. IEEE Trans. Dependable Secur. Comput..

[10] Engelberg A., Wool A. Classification of Encrypted IoT Traffic despite Padding and Shaping. Proceedings of the 21st Workshop on Privacy in the Electronic Society (WPES).

[11] Medury L., Robinson L., Kandah F. A Dynamic GAN-Based Obfuscation Approach Against Profiling Attacks. Proceedings of the 50th IEEE Conference on Local Computer Networks (LCN).

[12] Wan Z., Cho J.H., Lu C.T., Ji B., Moore T.J., Kamhoua C. (2025). Game-Theoretic and Machine Learning-Based Defensive Deception for Dependable and Secure Cyber-Physical Systems.

[13] Yang Y.T., Zhu Q. (2024). Game-Theoretic Foundations for Cyber Resilience Against Deceptive Information Attacks in Intelligent Transportation Systems. arXiv.

[14] Kong R., Chen H. (2025). DeepCRF: Deep Learning-Enhanced CSI-Based RF Fingerprinting for Channel-Resilient WiFi Device Identification. IEEE Trans. Inf. Forensics Secur..

[15] Amamra A., Anunwah J.C., Louafi H. (2025). IoT Device Fingerprinting via Frequency Domain Analysis. Electronics.

[16] Alyami M., Alghamdi A., Alkhowaiter M.A., Zou C., Solihin Y. (2023). Random Segmentation: New Traffic Obfuscation against Packet-Size-Based Side-Channel Attacks. Electronics.

[17] Apthorpe N., Huang D.Y., Reisman D., Narayanan A., Feamster N. (2019). Keeping the Smart Home Private with Smart(er) IoT Traffic Shaping. Proc. Priv. Enhancing Technol..

[18] Brahma J., Sadhya D. (2022). Preserving Contextual Privacy for Smart Home IoT Devices With Dynamic Traffic Shaping. IEEE Internet Things J..

[19] Xiong S., Sarwate A.D., Mandayam N.B. (2022). Network Traffic Shaping for Enhancing Privacy in IoT Systems. IEEE/ACM Trans. Netw..

[20] Narayanan S. (2025). Software-Optimized Dynamic Traffic Padding for Enhancing Privacy in Smart Home IoT Networks. Int. J. Inf. Technol..

[21] Juarez M., Imani M., Perry M., Diaz C., Wright M. (2016). Toward an Efficient Website Fingerprinting Defense. Proceedings of the 21st European Symposium on Research in Computer Security (ESORICS), Heraklion, Greece, 26–30 September 2016.

[22] Zhou J., Du Y., Ma L., Shen Z., Criswell J., Walls R.J. Zero-Delay Lightweight Defenses against Website Fingerprinting. Proceedings of the 29th USENIX Security Symposium.

[23] Holland J.K., Hopper N. (2022). RegulaTor: A Straightforward Website Fingerprinting Defense. Proc. Priv. Enhancing Technol..

[24] Huertas Celdrán A., Sánchez Sánchez P.M., von der Assen J., Schenk T., Bovet G., Martínez Pérez G., Stiller B. (2024). RL and Fingerprinting to Select Moving Target Defense Mechanisms for Zero-Day Attacks in IoT. IEEE Trans. Inf. Forensics Secur..

[25] Sánchez Sánchez P.M., Huertas Celdrán A., Bovet G., Martínez Pérez G. (2024). Adversarial Attacks and Defenses on ML- and Hardware-Based IoT Device Fingerprinting and Identification. Future Gener. Comput. Syst..

[26] Granados A., Miah M.S., Ortiz A., Kiekintveld C. (2020). A Realistic Approach for Network Traffic Obfuscation Using Adversarial Machine Learning. Decision and Game Theory for Security.

[27] Verma G., Ciftcioglu E., Sheatsley R., Chan K., Scott L. Network Traffic Obfuscation: An Adversarial Machine Learning Approach. Proceedings of the IEEE Military Communications Conference (MILCOM).

[28] Hu Z., Liu L., Gong J., Zhang Y., Guo M., Ge M., Guo Q., Ma L., Yu X. (2025). TOPLDM: Towards Dynamic Low Overhead Traffic Obfuscation Based on Packet Length Distribution Modification. Comput. Netw..

[29] Xiao F., Huang Y., Zuo Y., Kuang W., Wang W. (2023). Over-the-Air Adversarial Attacks on Deep Learning Wi-Fi Fingerprinting. IEEE Internet Things J..

